# Computational Modeling of Interventions for Developmental Disorders

**DOI:** 10.1037/rev0000151

**Published:** 2019-06-06

**Authors:** Michael S. C. Thomas, Anna Fedor, Rachael Davis, Juan Yang, Hala Alireza, Tony Charman, Jackie Masterson, Wendy Best

**Affiliations:** 1 Developmental Neurocognition Lab, Birkbeck, University of London; 2 MTA-ELTE Theoretical Biology and Evolutionary Ecology Research Group, Budapest, Hungary; 3 Developmental Neurocognition Lab, Birkbeck, University of London; 4 Department of Computer Science, Sichuan Normal University; 5 Developmental Neurocognition Lab, Birkbeck, University of London; 6 Institute of Psychiatry, Psychology, and Neuroscience, King’s College London; 7 Department of Psychology and Human Development, UCL Institute of Education, University College London; 8 Division of Psychology & Language Sciences, University College London

**Keywords:** computational modeling, connectionism, developmental disorders, intervention

## Abstract

We evaluate the potential of connectionist models of developmental disorders to offer insights into the efficacy of interventions. Based on a range of computational simulation results, we assess factors that influence the effectiveness of interventions for reading, language, and other cognitive developmental disorders. The analysis provides a level of mechanistic detail that is generally lacking in behavioral approaches to intervention. We review an extended program of modeling work in four sections. In the first, we consider long-term outcomes and the possibility of compensated outcomes and resolution of early delays. In the second section, we address methods to remediate atypical development in a single network. In the third section, we address interventions to encourage compensation via alternative pathways. In the final section, we consider the key issue of individual differences in response to intervention. Together with advances in understanding the neural basis of developmental disorders and neural responses to training, formal computational approaches can spur theoretical progress to narrow the gap between the theory and practice of intervention.

In this article, we consider the application of connectionist networks to modeling interventions to remediate developmental deficits, focusing on disorders of speech, language, communication and literacy. Recent connectionist models have made progress in simulating patterns of *acquired* deficits by incorporating neuroanatomical constraints into their architectures. For example, in Ueno et al.’s (2011) model of the reading system, a dual pathway model of reading was constrained to include the ventral and dorsal anatomical routes linking primary auditory cortex to motor cortex, and was able to simulate patterns of acquired deficits in word repetition, word comprehension, and word naming. Chen, Lambon Ralph, and Rogers’ (2017) model of the semantic system employed a spoke and hub architecture, constrained by the heteromodal integrative function of anterior temporal lobe, the hub linking representations of concepts in different sensory and motor systems, and was able to capture patterns of deficits in semantic dementia and visual agnosia in picture naming (see also Hoffman, McClelland, & Lambon Ralph, 2018). These models simulated deficits by removing connections from certain regions or pathways in their architectures guided by cognitive neuroscience data, and were then able to simulate patterns of recovery by relearning in the impaired model. In some cases, the effects of interventions were considered by altering patterns of subsequent retraining (e.g., Plaut, 1996, 1999). Modeling of *developmental* disorders, however, is less advanced, to date mainly focusing on single network models of individual abilities. Nevertheless, because such models focus on mechanisms of change as a cause of disorders, they offer a good foundation to consider interventions.

Developmental disorders differ from acquired disorders, in that the cause of the deficit is not removal of structures supporting established functionality but a developmental process that occurs under atypical constraints. The developmental process is characterized by complex and interacting cascades, by effects of timing, and by plasticity that affords opportunities for compensation (Karmiloff-Smith, 1998, 2009; Thomas & Karmiloff-Smith, 2002; Woollams, 2013). Over 30 years, a range of developmental connectionist models has advanced explanations for behavioral deficits in disorders such as dyslexia, developmental language disorder (DLD), autism, and attention-deficit/hyperactivity disorder (ADHD) (see Mareschal & Thomas, 2007; Thomas, Baughman, Karaminis, & Addyman, 2012; Thomas & Karmiloff-Smith, 2003a, for reviews). Having established this foundation, some authors foresaw an influential role for connectionism in intervention research. Daniloff (2002, p. viii) argued that connectionism “will . . . form the backbone of much of language therapy in the near future,” whereas Poll (2011, p.583) argued that “insights from connectionist research on the acquisition of early morphology and syntax can provide theoretical guidance for language intervention.” Despite the enthusiasm, this potential has yet to be realized, with very few models of developmental deficits being extended to address behavioral interventions (see Best et al., 2015; Harm, McCandliss, & Seidenberg, 2003, for exceptions).

One should not see this as a failure of connectionist approaches per se. The gap between theories of deficit and theories of intervention is a more general phenomenon (see e.g., Michie & Prestwich, 2010). To take one example, developmental disorders of language, it has been argued that despite extensive theories about the causes of behavioral deficits, such theories have played a relatively small role in the intervention practices of speech and language therapists; and indeed, theories of treatment have often developed relatively independently of theories of deficit (Law, Campbell, Roulstone, Adams, & Boyle, 2008). There are multiple reasons for the gap. These include (a) the complexity of the intervention situation, which involves treatment of the whole child via a social interaction with the therapist, and where the techniques employed are often dependent on the characteristics of the individual child, their response to intervention, and the therapist’s experience and intuitions; (b) the diverse real-world constraints on interventions, including resources like time and cost; (c) the primary focus of intervention on behavioral outcomes, which do not in themselves necessitate an understanding of cause; (d) frequent lack of an evidence-based consensus on the most effective treatment for a given deficit; (e) the fact that children often do not have a single ‘deficit’ either behaviorally or in terms of underlying mechanisms; and (f) even when a theory of deficit exists, the difficulty of moving straightforwardly from that theory to a prediction of best treatment. As Byng (1994) argued, although theories of deficit are a necessary precursor to developing interventions, “simply having a detailed analysis of the deficit does not by itself suggest the formulation of specific therapeutic procedures to effect change” (p.270). What is needed is a theory of intervention.

In what follows, we review contributions from existing connectionist models and our own work to assess whether any general principles of intervention can be identified from this approach. The following broad principles will emerge: the exact nature of the computational deficit matters for the success of intervention, as does its location in more complex architectures; the timing of the intervention matters, and its content with respect to the target behavior; computational methods have not revealed ways to trigger new engagement of compensatory mechanisms; as yet relatively unexplored are the implications of dosage, duration, intensity and regimes of behavioral interventions, and how to ensure both generalization beyond training items and persistence of intervention effects. In the following sections, we characterize the nature of the intervention process, to establish the challenge of building a computational model of how this process may act on cognitive mechanisms; we summarize how developmental disorders are captured within connectionist approaches; and we outline two previous models of interventions, for dyslexia and for word-finding difficulties.

## The Intervention Process: The Example of Behavioral Interventions for Developmental Disorders of Language

*Intervention* is a broad term that encompasses a wide range of activities. One definition, in the context of improving the language skills of children with speech, language, and communication needs, describes an intervention as “an action or technique or activity or procedure (or indeed combinations of these) that reflects a shared aim to bring about an improvement, or prevent a negative outcome . . . this can also include the modification of factors that are barriers or facilitators to change and the modification of an environment to facilitate communication development” (Roulstone, Wren, Bakopoulou, & Lindsay, 2012, p. 327). Roulstone et al. identify several terms that are sometimes used interchangeably, including *treatment*, *therapy*, *intervention*, and *remediation*.

One principal determining factor influencing choice of intervention method is the child’s age. *Implicit* techniques are employed with younger children, whereas *explicit* techniques are frequently employed with older children (Law et al., 2008; Stokes, 2014). For younger children (less than 6 years), the main aim is skill acquisition. Techniques are informal and naturalistic, with implicit goals and methods embedded in child-directed learning contexts. For older children (more than 6 years), intervention also targets metacognitive abilities and the development of compensatory strategies. There is greater use of formal methods, employing explicit goals and instruction in a therapist-directed learning context. Although there is a general view that targeting causal processes early in disordered development may be more effective than waiting until outcomes are established (Wass, 2015), systematic evaluations of timing-of-intervention effects are less common. Important dimensions of the intervention method include the precise nature of the intervention itself; who delivers the respective components of the therapy (e.g., a speech and language therapist [SLT], an SLT assistant, a teaching assistant, teacher, parent, or a computer); if the therapy is delivered one-to-one, or in a group; and the dosage of the intervention, including intensity and duration (Ebbels, 2014).

To give a concrete example of an intervention in a specific domain, Seeff-Gabriel, Chiat, and Pring (2012) evaluated an intervention to improve performance in producing regular English past tenses for a 5-year-old child with speech and language difficulties. The intervention was delivered one-to-one by an SLT, with carryover from the mother and the school. Facilitation methods were used, including modeling and elicitation, to help the child produce the correct past tenses, combined with visual symbols to provide metalinguistic support. The intervention dose was 30 min a week for 10 weeks with the SLT for a total of 5 hr, plus the additional input from the mother and school. This pattern is representative of a single block of intervention: in a survey of over 500 SLTs in the U.K., Lindsay, Dockrell, Law, Roulstone, and Vignoles (2010) reported the most common frequency of delivery of a language therapy was once a week for 6 weeks or more, with 42% asking teachers and parents to deliver the intervention more frequently between visits to increase the dosage. Blocks may be repeated. This typical dose and duration can be contrasted with the much larger dosages sometimes used with other developmental disorders, for instance to address the wider sociocommunicative deficits in autism. In one form of the early intensive behavioral intervention (EIBI), intervention begins by 2 years of age, with a range of 20 to 40 hr per week across one to four years of the child’s life, for a range of intervention dose of between 1000 and 8000 hr (Eikeseth, 2009; Smith, 2010).

Children can vary widely in their response to interventions. Apart from the age of the child, other characteristics are relevant to intervention outcome, including the severity of the developmental deficit and the presence of other comorbid deficits (Ebbels, 2014). The relationship between dosage and the effect size of the behavioral improvement produced by the intervention also varies, and depends on the target ability. For example, Lindsay et al. (2010) summarized meta-analysis data to indicate that for interventions targeting phonology, intensive interventions were more effective than those of long duration; for those targeting syntax, interventions of long duration were more effective than short intensive ones; for vocabulary, long duration was important but not intensity—children did better with short bursts over an extended time. In a well-controlled study of a grammar treatment for 5-year-olds with DLD, Smith-Lock et al. (2013) found that the same dose of 8 hr was more effective delivered weekly over 8 consecutive weeks than daily over 8 consecutive days. Differences in optimal regimes presumably depend on the functional plasticity of the underlying mechanisms, including time for consolidation and opportunities for practice.

Approaches vary as to whether the primary aim of intervention is to remediate the deficit or to encourage development of potential compensatory strengths. To give an example, word-finding difficulties (WFD) represent a developmental vocabulary deficit where children struggle to produce words that they can nevertheless comprehend. WFD is viewed as a heterogeneous disorder, with possible causes either in phonological access or impoverished semantic representations (Best, 2005; Faust, Dimitrovsky, & Davidi, 1997). In a survey, Best (2003) reported that SLTs listed phonological awareness difficulties as co-occurring with WFD 46% of the time, whereas semantic problems co-occurred only 13% of the time. However, intervention approaches that targeted semantics were used more frequently than those that targeted phonology (79% of the time compared with 54%). In this case, therefore, SLTs often sought to buttress areas of strength within the child to improve word-retrieval skills.

The order of targeting skills within a domain may also be important. For example, in the usage-based approach to remediating developmental problems in syntax, grammatical structures are targeted in the same order that they develop in typically developing children (e.g., Riches, 2013); that order of acquisition reflects the interaction between the challenges of the particular domain and the constraints of developmental mechanisms.

A key question is which intervention the therapist should choose. The decision is influenced by multiple factors. A key factor, of course, should be the intervention’s effectiveness. However, Roulstone et al. (2012) noted that evidence for effectiveness incorporates clinical experience or local evaluations, in addition to research evidence. Roulstone et al. identified several other factors influencing intervention choice, including reference to underlying theoretical positions, and pragmatic reasons related to efficiency, accessibility, popularity, and cost. Other researchers have taken a wider perspective on the factors influencing the design and success of interventions aiming to change behavior. For example, Michie and colleagues (e.g., Michie, van Stralen, & West, 2011) constructed a framework that incorporates not just the internal cognitive mechanisms able to deliver behavioral change (which they termed *capability*), but also motivation and opportunity to change. The framework identifies environmental influences and structures, such as resources and policy, which operate as constraints on or incentives for success.

There are two important dimensions in the evaluation of interventions. The first is the extent to which the intervention *generalizes* to other items or skills beyond those targeted in the intervention itself. The second is the *persistence* of the benefits of intervention after the intervention has ceased. Using our example study of Seeff-Gabriel et al. (2012) that targeted English past tense, the 5-hr intervention was found to generalize to untrained regular verbs but not to other irregular verbs, while progress was maintained at follow-up 8 weeks later. Generally, achieving generalization and persistence of interventions has proved challenging. For example, in her review of interventions for grammar difficulties in school-age children, Ebbels (2014) concluded that follow-up generally shows that the progress produced by the intervention is maintained, but does not prompt acceleration in development after the intervention has ceased. The gains are retained but no further gains are stimulated. Bailey, Duncan, Odgers, and Yu (2017) identified the diminishing effect of an intervention after its cessation (so-called *fade-out*) as a characteristic of many interventions targeting cognitive and socioemotional skills and behaviors.

Other important factors include: (a) the child preferences (e.g., a child’s willingness to work on target A but not B); (b) parental involvement (what are appropriate activities for home practice to maximize dose); (c) context (e.g., selecting vocabulary items to mirror those currently being taught in the school curriculum); and (d) outcome of intervention (such that the therapist may modify targets, methods, and feedback according to the response to intervention).

Lastly, even if an intervention has been shown to be effective, unless its key active ingredient has been understood, it is not guaranteed that the effect will be similar when applied to new children, when delivered by less expert practitioners, or when adapted to new contexts. Identification of the active ingredient in turn is facilitated by comparison to a control group whose treatment differs only in the active ingredient. And this in turn requires a theory about how the intervention remediates the deficit or supports a compensatory strategy.

In summary, this concrete example of interventions for developmental disorders of language illustrates the complexity of the process and the multiple factors involved. Interventions involve activities to improve developmental outcomes in children, outcomes are variable depending on the characteristics of the child and therapist, both the design and the dosage of the intervention are important for outcome, and outcomes need to be evaluated against key criteria of (a) generalization to other items or skills beyond those targeted in the intervention itself and (b) maintenance of gains once the intervention has ceased.

## Connectionist Models of Interventions

### How Disorders Are Simulated: Monogenic Versus Polygenic Approaches

In theory, the recent neuroanatomically constrained connectionist models of the language system (Chen et al., 2017; Ueno et al., 2011) lend themselves readily to simulating developmental deficits, via initial restrictions to the pathways or mechanisms taken to underlie a given behavior. For example, Seidenberg (2017) summarized recent cognitive neuroscience hypotheses that developmental dyslexia may be the result of four types of deficit: anomalies in myelinization affecting the speed and reliability of signal transmission within and between reading/language areas of the brain, neuronal hyperexcitability within areas, anomalies of neural migration impacting the functionality of neural networks, and increased variability/noise in neural representations impacting the functional connectivity between regions of the reading network and the ability of the system to benefit from learning experiences (see also Hancock, Pugh, & Hoeft, 2017). Much of the existing work on developmental disorders, however, has focused on connectionist models of individual mechanisms acquiring single target behaviors. In this latter work, a distinction can be drawn between *monogenic* and *polygenic* models of disorders.

In a single network model, changes in behavior are the result of experience-dependent alterations to the structure of the network, caused by its interaction with a learning environment with particular informational content. Artificial neural networks have intrinsic constraints that affect what input–output mappings they can learn and how quickly. These constraints include properties such as the number of internal (hidden) units, the pattern of connections between units, the rate at which connection strengths change in response to experience, and the way external or environmental inputs are encoded for processing. Models of developmental deficits propose that these constraints are atypical in some children, deflecting developmental trajectories outside the normal range of variation (Thomas & Karmiloff-Smith, 2003a, 2003b). For instance, in an early model of developmental dyslexia, the deficit was simulated by attempting to learn the mappings between orthography and phonology in a model with too few hidden units (Seidenberg & McClelland, 1989); in a model of autism, overdetailed categories were simulated by increasing the number of hidden units in a semantic network (Cohen, 1994).

The Seidenberg and McClelland model of dyslexia (1989; see also Harm & Seidenberg, 1999; Plaut, McClelland, Seidenberg, & Patterson, 1996) illustrates what might be called the *monogenic* approach. Connectionist models usually have several free parameters, such as the number of internal or hidden units, the learning rate, and the momentum. Values for these parameters are determined so that the model captures the trajectory of typical development. In the disordered case, *just one parameter is set to a different value*. The disorder, then, has a single cause, against a background of very small or zero variation in all other computational parameters across individuals (Thomas, 2003).

More recent models have adopted a *polygenic* approach (e.g., Thomas, Forrester, & Ronald, 2016; Thomas & Knowland, 2014; Thomas, 2016a). Individual variation in the development of cognitive abilities is viewed as arising from the combined influence of small variations in many neurocomputational parameters, including those involved in the construction, activation dynamics, adaptation, and maintenance of network architectures. The approach involves simulating development in large populations of individuals. The cumulative effect of many small contributions produces a normal distribution of the development of behavior in the population, against which a normal range of variation can be defined, and cases of developmental delay identified (Thomas, 2016b). Disorders are thus viewed as the lower tail of a continuous distribution of developmental variation in a population.

The *monogenic* and *polygenic* approaches are not mutually exclusive. For example, Thomas and colleagues demonstrated how autism might combine two groups, monogenic cases with a genetic mutation causing a given neurocomputational parameter to take up extreme values, and polygenic cases with the same parameter falling in the upper normal range but having its effect on behavior amplified by a combination of risk factors that vary across the whole population (Thomas, Davis, et al., 2016; Thomas, Knowland, & Karmiloff-Smith, 2011; see Leblond et al., 2019, for recent genetic results). Furthermore, interaction of a monogenic cause and population-wide polygenic individual differences can give rise to apparent subgroups within the developmental disorder despite it having a single pathological cause: individual differences that produce small effects normally can be exaggerated by the atypical parameter, causing divergent manifestations of the disorder (Thomas, Davis, et al., 2016; Thomas, 2016b).

### How Interventions Are Simulated

Where a developmental deficit is identified in a child, it is presumed that naturalistic experience (or typical educational experience) has not been sufficient to enable the emergence of age-appropriate behaviors. In a single network model, two types of intervention are suggested: the additional of new information to the structured learning environment (in simulation terms, new/replacement patterns in the training set); or manipulations to the computational properties of the system (equivalent, say, to pharmacological treatments, transcranial magnetic stimulation, or neurofeedback). In some types of models, changes in computational properties might subsequently serve to alter the system’s sampling of its learning environment (such as in reinforcement learning models; e.g., Richardson & Thomas, 2006). In a model that simulates a range of behaviors in a larger architecture, such as in a full reading system, the possibility exists not only of intervening to remediate atypical mechanisms/pathways, but also to exploit pathways without atypical processing constraints. As we saw previously, actual interventions vary as to whether they target remediation of deficit or support of compensatory strengths, perhaps depending on the severity of the deficit (Woollams, 2013). However, the exact nature of the interaction between processing components may be important in understanding the effects of either type of intervention.

How could one select further training items—an intervention set—for an atypically developing network, which would be more successful in driving development than natural experience? The statistical learning perspective of which connectionism is a part has generated a growing understanding of environmental factors that produce stronger or weaker learning in typical development (Borovsky & Elman, 2006; Gomez, 2005; Onnis, Monaghan, Christiansen, & Chater, 2005). This includes the importance of factors such as the frequency of training items, their variability, and the provision of novelty in familiar contexts. For example, one heuristic that arises from this approach is that to improve acquisition of *compositional* domains, where concepts are made up of different combinations of the same primitives, the system should be exposed to the component primitives, either in isolation or in many different combinations (see, e.g., Fey, Long, & Finestack, 2003). This also encourages subsequent generalization to novel instances. Potentially, these kinds of lessons can provide guidance on how to design intervention sets to achieve the best behavioral outcome for a model with atypical computational constraints. However, this would be to assume that an understanding of the experiences that improve or hinder learning in *typically* developing systems is informative about how to influence developmental outcomes in cognitive systems with *atypical* constraints (an assumption that drives, for example, the usage-based approach for grammar deficits; Riches, 2013). If principles of typical development are a guide, connectionist approaches to language acquisition highlight several factors (Poll, 2011): that the structure and quantity of the input is important in driving development, that language development does not occur through passive exposure but via experiences related to the child’s own expectations, and that language development concerns learning the relationship between language form and language meaning so that contextual cues which narrow the hypotheses will aid learning. However, it remains to be demonstrated in implemented models that the factors producing best development in typical models also hold for those with atypical processing properties.

Cognitive computational models point to an important distinction between two types of behavior in evaluating interventions. The first is *performance on the training set*, that is, the range of experiences the system encounters in its structured learning environment. The second is *performance on a generalization set*, that is, items which are novel to the system but which bear similarity to those with which it has experience. This echoes the concern in actual interventions on whether the intervention *generalizes* to other items or skills beyond those targeted in the intervention itself. Computational systems with a so-called inductive bias (Mitchell, 1997) can take advantage of their existing knowledge to produce responses to novel inputs. If—externally, as modelers—we stipulate that the structured learning environment in fact contains some underlying regularity or function, we can assess the generalization performance of a system depending on whether it has extracted this underlying function from its training examples, and is then able to apply it appropriately to novel items. In models, the distinction between training and generalization is important because developmental deficits may operate differentially across performance on the training set and the generalization set, because actual interventions are often assessed specifically on their ability to produce generalization beyond the treated items, and because interventions can be chosen which differentially target training set or generalization performance.

Two previous models have given serious consideration to the use of models of atypical development (respectively, in dyslexia and in word-retrieval difficulties) to evaluating potential interventions. Harm et al. (2003) extended the triangle model of reading (Harm & Seidenberg, 1999; Plaut et al., 1996; Seidenberg & McClelland, 1989) to address an apparent paradox that, although a phonological deficit is often viewed as the primary cause of developmental dyslexia, interventions that target spoken language (phonology) alone are relatively ineffective at remediating reading deficits once a child has learnt to read. Instead, interventions to facilitate reading aloud need to combine work on phonology and on decoding, that is, learning the mapping between print and sound (Bus & van Ijzendoorn, 1999). Harm et al.’s (2003) model of reading involved a phonological component, which first learned a lexicon of English words. An orthographic component then provided representations of the written forms of words, which had to be associated with the existing phonological representations. Dyslexic versions of the model were produced by applying atypical constraints to the phonological component, which impacted on its initial phase of acquisition. Specifically, prior to training, 50% of the connection weights were set to and held at zero, and weight decay was applied to the remaining weights, thereby limiting the maximum magnitude that they could reach during training. Before reading acquisition commenced, phonology was atypical. The outcome of reading acquisition was a system with a particular deficit in its nonword reading, that is, its generalization of reading to novel forms. Such a deficit has been termed phonological developmental dyslexia (Castles & Coltheart, 1993).

Harm et al. (2003) then compared two interventions, each applied at two different points in training. One intervention simply alleviated the phonological deficit—unfroze the 50% of weights and removed weight decay. One could view this as an intervention that directly targeted neurocomputational properties. The second intervention added new items to the training set, to simulate a particular behavioral intervention (the Word Building Intervention; McCandliss, Beck, Sandak, & Perfetti, 2003). This took the form of extra lessons on an ordered sequence of words each of which differed by changing or moving only one grapheme (e.g., sat, sap, tap); where the model made an error, extra training was given on the individual component grapheme-phoneme mappings of a word (for “sat,” s → /s/ in first position, a → /a/ in second position, etc.). Both interventions produced benefits to nonword reading, albeit without fully remediating the deficit to the levels observed in the typically developing model. The timing of intervention was also important. Alleviating the phonological deficit alone only showed benefits when applied early in training, whereas the simulated behavioral intervention that targeted decoding showed benefits across training. The explanation for this *age-related* effect, paralleling the observed empirical data, was that once the network began to learn mappings between orthography and impoverished representations of phonology, these were hard to undo even if phonology was remediated later on. An apparent sensitive period for remediation by training phonology alone, therefore, was explained by *entrenchment*: the difficulty of resetting inappropriately configured connection weights (Thomas & Johnson, 2006). Viewing Harm et al.’s (2003) model as representing two components in the larger reading architecture (Ueno et al., 2011), these timing effects speak to the importance of understanding the developmental interactions between multiple components with the architecture.

In this model, then, the initial developmental deficit was mainly in generalization rather than performance on the training set. The deficit was remediated by showing the network the component parts of holistic representations (in line with the heuristic identified in statistical learning approaches) through the particular sequence of presentation of items in the lesson, and the addition of new information to the training set in the form of individual grapheme-phoneme correspondences. Lastly, there was a contrast between an intervention that directly targeted computational properties, and a behavioral intervention, which added something new to the training set and/or changed the frequency distribution within the training set.

The second model by Best et al. (2015) explored interventions for children with word-finding difficulties (WFD). Naming was implemented as the activation of a semantic representation of the word’s meaning activating its phonological form. Developmental deficits in productive vocabulary may be caused in at least two ways: by impairments in the semantic representations driving naming, or by impairments in accessing phonological output forms. Evidence suggests remediation of both semantic and phonological knowledge can produce benefits for these children (Best et al., 2017). The connectionist naming model had two components: a semantic component and a phonological component, each of which underwent its own developmental process to establish its internal representations; and two pathways to learn the mappings between these representations as they developed, from semantics to phonology to simulate naming, and from phonology to semantics to simulate comprehension. Constraints applied to either of these components, or to the pathways between them, produced developmental naming deficits. The model was used to predict the outcome of interventions on two individual 6-year-old children diagnosed with WFD. Two atypical models were calibrated to resemble the developmental profiles of the individual children, according to measures of the children’s phonological knowledge, semantic knowledge, naming, and comprehension abilities. The model manipulations involved removing connections, reducing the number of hidden units, or altering the activation dynamics of the simple processing units, either in the components or the pathways, but always prior to training.

The individual models were then given either a semantic or a phonological intervention. The semantic intervention involved additional training for the semantic component to improve its internal representations, whereas the phonological intervention involved additional training for the phonological component. The interventions were interleaved with the normal training regime for vocabulary development. The result was a prediction for which type of intervention would work best for each child. The model predictions were then tested in reality by giving each child both a semantic and a phonological intervention in turn (one session of 30 min per week for 6 weeks, for a total of 3 hr for each intervention type, and a 6-week wash-out period between interventions). It was then determined which intervention improved naming skills more. For one child, the model’s prediction was correct (only the phonological intervention benefited naming performance); for the other child it was not (the model predicted both interventions would work; the child only benefited from the semantic intervention).

In this model, a behavioral intervention was again simulated by modifying the training set, here altering the relative amount of training on different components of the system, but without the addition of new information. Intervention success was measured against performance on the training set, although the intervention occurred only on a subset of the full training set. The model focused on differential effects of intervention type and did not report whether deficits were fully remediated in either case.

Table 1 summarizes some of the key concepts identified in the introduction.[Table-anchor tbl1]

## Outline of Modeling

We review an extended program of modeling work (see author note), in four sections. In the first, we consider *long-term outcomes*. Developmental disorders are diagnosed in childhood, when a child is flagged as not meeting age-appropriate performance expectations. Computational models allow consideration of the long-term outcomes, if these systems are left to develop without interventions. We ask: (a) in the absence of intervention, what compensatory outcomes can be reached? And (b) do some early delays resolve, and if so under what conditions? In the second section, we address *methods to remediate atypical development in a single network*. We consider (a) where the disorder arises through insufficient early stimulation of the target system; (b) how to choose better training items to achieve learning in a system with atypical processing properties; (c) how better performance can be achieved from an atypical network by targeting improvement of its input and output representations; and (d) how interventions might instead alter the computational properties of the learning system. In the third section, we address *interventions to encourage compensation via alternative pathways*. In the final section, we consider the key issue of *individual differences in response to intervention*.

## Computational Modeling

### Simulating the Long-Term Outcome of Atypical Development Without Intervention

#### Compensated outcomes

An implemented model of a developmental deficit provides the foundation to investigate different possible interventions applied in childhood. But the modeler can also refrain from intervening, and use the model to predict the ultimate developmental outcome. For some computational limitations, sufficient exposure to the training set eventually permits performance to reach the normal range on this set. However, close inspection of these networks indicates that the underlying processing itself has not normalized. This can be demonstrated by observing a persisting deficit on generalization. Such an effect was observed in a connectionist model of English past tense formation simulating children with DLD.

The model of Thomas (2005) explored the theoretical proposal of Ullman and Pierpont (2005) that children with DLD might have a particular deficit in morphosyntax because of a more general deficit in their procedural memory systems. The so-called procedural deficit hypothesis addressed the observation that children with DLD often exhibit greater impairment in grammar development than vocabulary development. According to the hypothesis, the disparity stems from a differential reliance of the normal language system on two separate, more domain-general memory systems: grammar development on the procedural memory system, whose characteristics are slow acquisition, fast automatic execution, and sequence processing, and vocabulary development on the declarative memory system, whose characteristics are parallel processing and slow recall. Notably, the hypothesis proposed a central role for compensation in explaining observed behavioral impairments in DLD: the profile of language skills is a consequence of the procedural system’s suboptimal attempts to acquire the structural aspects of language *combined* with the attempts of the declarative memory system to compensate for this shortcoming through lexical strategies.

**Figure 1 fig1:**
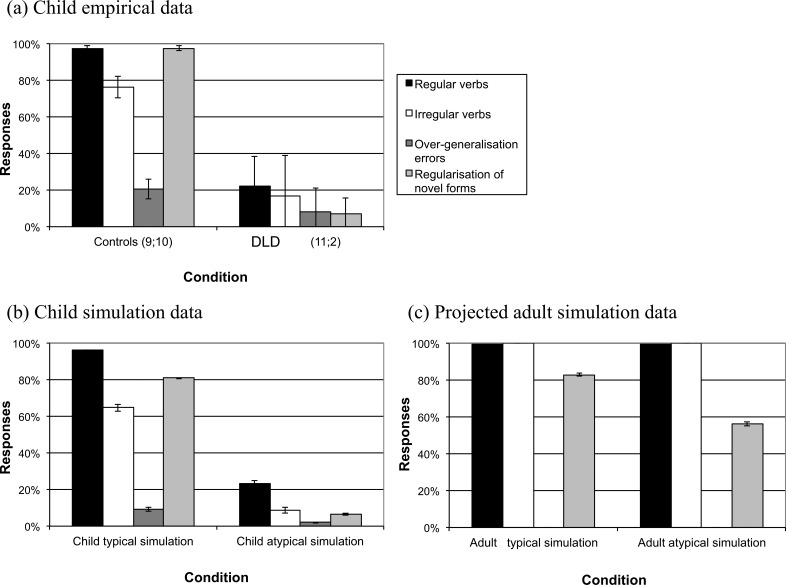
Simulation of typical and atypical past tense acquisition predicting long-term compensated outcomes. (a) Empirical data (per cent accuracy) for typically developing children from Thomas et al. (2001) for a group of typically developing children on a past tense elicitation task for regular verbs, irregular verbs, novel verbs, and overgeneralization errors; and for a group of children with DLD from van der Lely and Ullman (2001), using the same elicitation task. Error bars show standard error of the mean. (b) Simulation data from Thomas (2005) for a connectionist past tense model, either in a typical condition or an atypical condition where the discrimination of the simple processing units was reduced by lowering the temperature of the sigmoid activation function (1 → 0.25). Model data are shown at a point that approximately matched the performance of the children (250 epochs of training). (c) Simulation data for the projected ‘adult’ outcome of typical and atypical trajectories (5000 epochs of training). The projected adult model reached ceiling on the training set but retained atypical generalization. Error bars show standard error over 10 replications with different initial random seeds.

Thomas (2005) explored this idea with a model of English past tense acquisition in which the production of phonologically encoded past tense forms at the output was driven by integrating lexical-semantic and phonological information about the verb presented at the input (Joanisse & Seidenberg, 1999). DLD was simulated as a *monogenic* disorder, altering the activation function in the internal processing units prior to training to decrease their discriminability, in line with more recent neural noise accounts of developmental language deficits (Hancock et al., 2017). Unit discriminability was reduced such that units were less able to make large changes in their output for small changes in their input, implemented by reducing the temperature parameter in the sigmoid activation function from 1 to 0.25. This impaired the network’s ability to form sharp categorical boundaries in its internal representations. Figure 1 demonstrates the match of model data to empirical data in a past tense elicitation task for children of 10–11 years of age, either with or without DLD. As well as capturing the profile of reduced accuracy, the model captured a key compensatory feature identified by Ullman and Pierpont in the inflection of regular verbs in children with DLD: increased frequency effects (2005; see van der Lely & Ullman, 2001). Ullman and Pierpont took these frequency effects to be a key hallmark of the operation of declarative memory rather than procedural memory and to reflect its unusual involvement in morphosyntax in DLD. The connectionist model also captured the compensatory hallmark. In the model, it was instantiated as a greater role for lexical information in driving past tense formation, rather than learning the phonological regularities relating base and inflected verb forms that capture the past tense rule in the emergentist account of acquisition. Removing lexical-semantic input in the DLD model impaired regular verb performance, but did not in the typically developing model.[Fig-anchor fig1]

Figure 1 now shows what happened when the atypical model was allowed to run to its adult state. Performance on the training set, on both regular and irregular verbs, reached ceiling. Notably, however, there was a residual deficit on generalization, the extension of the regular past tense rule to novel forms. The model, with its atypical processing properties, had not managed to extract the general function within the training set, but with enough exposure to the training set, had eventually managed to produce normal-looking behavior on that set. Even in the adult state, the atypical network relied more on lexical information at input to drive its inflections.

Reducing the discriminability of processing units particularly impacted on generalization because it affected the formation of sharp category boundaries. Categorical functioning allows novel forms to be treated in the same way as existing category members. In unpublished work, the simulations reported in Thomas (2005) were run with other *monogenic* causes of the initial deficit. For two other deficits, processing noise and a purely lexical strategy for producing inflections, a similar pattern was observed of resolving delay on the training set and a residual generalization deficit; for restricted numbers of internal processing units, there was a residual generalization deficit but also no resolution of the early deficit on the training set; for a very slow learning rate, there was no generalization deficit but a residual deficit in irregular verb performance within the training set. It is evident, then, that the nature and possibility of long-term compensatory avenues within this single mechanism model *were sensitive to the type of initial processing deficit*.

In one sense, one might view long-term deficits in extracting regularities in the problem domain as examples of a well-known characteristic of suboptimal artificial neural networks: overfitting the training data. We wish to emphasize an alternative view, however: that atypical processing properties may still allow some parts of the problem domain to be acquired with enough training. Another aspect of language and another type of neural network architecture illustrate this point. Thomas and Redington (2004) used a simple recurrent network to investigate the impact of atypical processing constraints on syntax processing. Given sufficient training, they observed that simple recurrent networks with atypical sequence processing properties could eventually find compensatory solutions in classifying syntactic constructions, but only for those constructions that could be comprehended via locally available lexical cues, not those relying solely on sequencing information for decoding.

In sum, a system that exhibits early delays through atypical processing properties may be forced through massive exposure to show normal-looking behavior on the training set—the items that are intensely practiced. However, this does not normalize processing properties. Residual deficits may remain, such as in generalization or in more demanding aspects of the task. This pattern of eventual good accuracy on practiced items along with subtle residual deficits is observed in some developmental disorders. For example, large dosages of reading experience can sometimes remediate reading accuracy deficits in dyslexia, but residual deficits can be found in reading speed and in spelling, both of which suggest the internal representations have not been normalized (Hulme & Snowling, 2009). These deficits may even be subtle: Leong, Hämäläinen, Soltesz, and Goswami (2011) found that highly compensated adults with dyslexia (undergraduate students at the University of Cambridge) showed significantly lower sensitivity to syllable stress than adults without dyslexia.

#### Resolution of early delays

Sometimes, for a subset of children, early observed developmental deficits can resolve apparently of their own accord. The resolution of deficits has been reported in several developmental disorders, including language (e.g., Dale, Price, Bishop, & Plomin, 2003), autism (e.g., Charman, 2014b; Fein et al., 2013), and ADHD (e.g., Biederman, Petty, Evans, Small, & Faraone, 2010) and has generated theoretical debate in each case. What does resolution of delay imply about underlying cause?

Thomas and Knowland (2014) used the same connectionist model of past tense acquisition as Thomas (2005) to investigate why early identified delay sometimes resolves. They argued that limitations in the plasticity of developmental mechanisms can initially produce similar behavioral patterns as limitations in computational capacity. Systems with limited plasticity require more exposure to learning events to produce an equivalent improvement in performance. Mechanisms exhibiting early delays through limited plasticity should therefore respond to interventions that simply enrich the level of naturalistic experience. Such systems should remediate to the normal range just through greater practice, without requiring specially designed interventions.

Unlike the Thomas (2005) model of past tense formation, Thomas and Knowland (2014) took a *polygenic* approach to language delay. Variation in rates of development was modeled in a large population of simulated children (*N* = 1000). Variation was caused by simultaneous small differences in 14 computational parameters, as well as in the richness of the language environment in which the child was raised. The computational parameters influenced properties of the learning mechanism such as network construction (e.g., number of internal units), network activation (e.g., unit discriminability, processing noise), network adaptation (e.g., the learning algorithm, the learning rate), and network maintenance (e.g., the level of pruning to eliminate unused connectivity, weight decay).^1^ Across the 14 parameters, Thomas and Knowland identified four broad types of *processing role* that parameters might serve. These roles were *capacity*, *plasticity*, *signal*, and *regressive events*. Parameters contributing to capacity influence the potential dimensionality of learned representations, and include the number of units and connections; for plasticity, contributing parameters modulate the size of the weight changes produced by experience; for signal, it is noise added to unit activations or thresholds for driving behavioral responses; for regressive events, it is parameters influencing maintenance of connectivity, such as pruning and weight decay. Some parameters contribute mainly to one role, such as number of processing units and denseness of connectivity contributing to capacity. Other parameters contribute to more than one role: the nature of the learning algorithm determines both what can be learned and also how quickly; the unit discriminability influences the quality of the signal propagating through the network but also modulates the rate of connection changes and therefore plasticity. A system with low *capacity* has a reduced ability to learn complex information, one with low *plasticit*y requires more experience to learn, one with poor *signal* struggles to acquire an accurate rendition of knowledge, while one with *regressive events* will lose plasticity and potentially knowledge across development.

**Figure 2 fig2:**
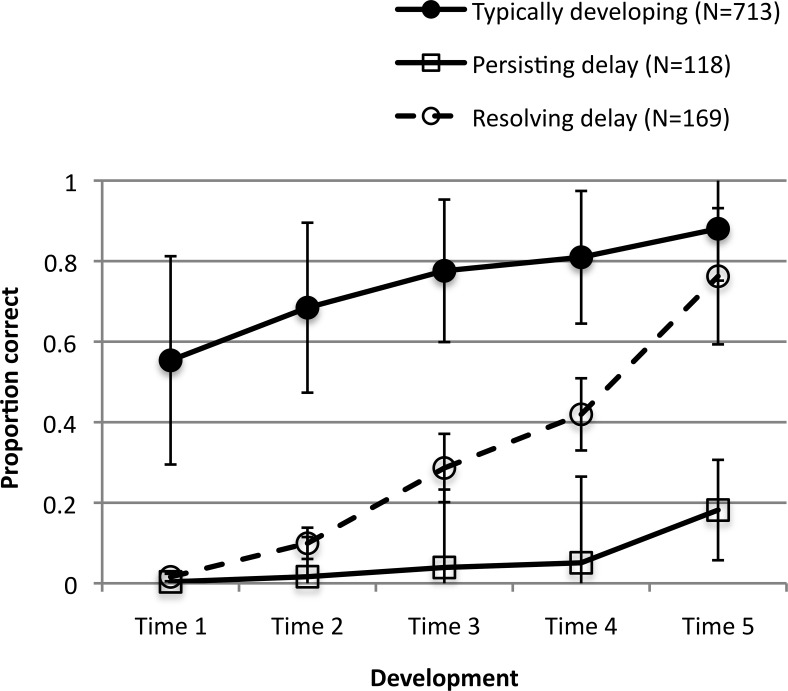
Simulation of resolution of early delay. Group averaged developmental trajectories for 1000 simulated children in a model of English past tense formation, assuming a polygenic model for language delay (Thomas & Knowland, 2014). Delay was defined at Time 1 as networks whose performance fell more than 1 standard deviation below the population mean. Networks were defined as having *resolving delay* if their performance fell within this normal range by Time 5; and as having *persisting delay* if their performance remained below the normal range by Time 5 (see Thomas & Knowland, 2014, for further details). Error bars show standard deviations.

Of the 1000 networks in the simulated population, 287 were diagnosed with language delay at an early point in development, based on falling 1 standard deviation below the population mean. The subsequent developmental trajectories of these delayed networks were followed, and 169 networks later resolved back into the normal range. Persisting deficits were observed in the remaining 118. Figure 2 shows the mean trajectories of the typically developing and delayed groups. The proportions are similar to those reported in the empirical literature, where early diagnosed language delay (e.g., aged 3–4) resolves in more than half of cases (e.g., by age 6; Bishop, 2005; Dale et al., 2003; see also Ukoumunne et al., 2012, for resolution at younger ages).[Fig-anchor fig2]

If the nature of intervention should be differentiated according to whether delay resolves or persists, it is important to be able to predict outcomes for children with early diagnosed delay as soon as possible (Chiat & Roy, 2008). However, researchers have found this challenging. For example, in a large empirical study, Dale et al. (2003) explored whether it was possible to predict if children would fall in the persisting delay (*n* = 372) or resolving delay (*n* = 250) group on the basis of their Time 1 profiles at 2 years of age, compared against Time 2 outcome at 4 years. Children whose delays would persist scored reliably lower across a number of parental rating measures, including vocabulary, grammar, displaced reference (use of language to refer to past and future events), and nonverbal skills, as well as scoring reliably lower for maternal education and showing a greater incidence of ear infection. Nevertheless, the effect sizes were small (.01–.06), and logistic regression analyses found that children’s profiles at age 2 offered only modest classification of outcome at age 4. The statistical regression model including vocabulary, displaced reference, and nonverbal scores at Time 1 correctly predicted only 45% of cases of persisting delay (chance = 50%), but 81% of cases of resolving delay. Addition of gender and maternal education level brought up the prediction of persisting delay to 52%.

A similar analysis was possible in Thomas and Knowland’s (2014) connectionist model. Here, Time 1 behavioral measures were broadly similar across persisting and resolving delay groups. There were subtle differences in past tense accuracy, with the persisting delay group performing reliably worse on regular verbs and generalization of the past tense rule to novel verbs (that is, in extracting the underlying regularities of the domain) compared with the resolving group. But although these effects were highly reliable, as with the empirical data, they were of small effect size. A logistic regression model entering just Time 1 behavioral profiles was 80% accurate in predicting persisting delay but only 54% accurate in predicting resolving delay. In the model, variations in the richness of the training environment implemented one pathway by which differences in maternal education have been proposed to influence language development (see Thomas, Forrester, & Ronald, 2013). Accuracy was not increased by adding in the richness of the language environment to which each network was exposed. As per Dale et al. (2003), measures of the environment didn’t help to predict developmental outcome. In one sense, this is quite surprising: in the model, experience of the language environment was the primary driver of development itself. Despite this central role, it was a weak predictor of individual differences.

Computational implementations provide the opportunity to investigate the mechanistic reasons why a model captures a given behavioral profile. In the current case, we can identify which of the computational parameters in fact predicted whether delay would resolve or not. Table 2 indicates which parameters had predictive power on developmental outcome. Limits on capacity tended to predict persisting delay, whereas limits in plasticity predicted resolving delay. When the full set of computational parameters was added into the logistic regression, a combination of Time 1 behavior and information about processing properties was able to predict persisting delay at 72% accuracy and resolving delay at 84%. (In clinical practice, 80% sensitivity and specificity is sometimes viewed as the requirement of a good screening test for developmental disabilities; it is less than 100% because clinical science is accepted as often imprecise; Charman et al., 2016; Glascoe, 1999.) It is notable that, in the model, sensitivity and specificity levels did not reach 100%. Failure to predict all the variance in outcome in a relatively simple and well-controlled model points to the complex dynamics involved in development of nonlinear learning systems.^2^ More importantly, the model suggested that to predict behavioral outcomes in cases of atypicality, measures of behavior need to be complemented with measures of processing, as argued by Fernald and colleagues (e.g., Fernald & Marchman, 2012).[Table-anchor tbl2]

Predictions derived from a computational model need to be mapped to cognitive or brain processes in the child. How do the properties of the model map to real children? Practically, capacity can be operationalized as the quantity of information that can be integrated online, such as in a phonological awareness task. Plasticity, by contrast, can be operationalized as performance on a learning task, such as in auditory statistical learning. The computational perspective suggests these properties are likely to be related but potentially distinguishable by focusing on change over time, either in experimental tasks or in longitudinal trajectories.

In sum, resolution of an early identified developmental deficit can occur if the atypicality in the system is a limitation in plasticity rather than capacity. In this case, natural experience may drive the resolution. The implication is that intervention need only increase the dosage of naturalistic experience, for example by encouraging more frequent language interactions in the home, rather than employ a specially designed intervention. However, identifying early on whether an emerging delay is attributable to a plasticity rather than a capacity limitation is challenging and requires attention to processing properties rather than just behavioral profiles and environmental measures.

### Simulating Methods to Remediate Atypical Development in a Single Network

From a computational standpoint, behavioral interventions seeking to ameliorate deficits can be construed as changing the experiences the system is exposed to, for example through a discrete block of intervention. This could either amount to reweighting of information available in previous experience, to blocked practice of certain skills, to alterations in salience or feedback; or it could be different experiences to those encountered before. The starting point is the assumption that naturalistic experience (or the usual range of educational experiences) has not been sufficient for the system to acquire age-appropriate abilities; and this is because the learning mechanism has atypical processing properties. If a system has limitations, why should adding further or different experiences improve the situation? Intervention might cause a beneficial restructuring of representations, and do so by using feedback or concentrated practice to emphasize certain dimensions or associations within the task domain. Of course, this is predicated on the assumption that the mechanism, and indeed the child more broadly, has indeed been exposed to the appropriate range of experiences prior to diagnosis of the disorder. We begin by considering the possibility that this is not the case.

#### Disorders from insufficient early stimulation

Although clinicians usually attempt to rule out environmental causes in diagnosing developmental disorders, language disorders are often observed with increased frequency in children from low SES backgrounds (All Party Parliamentary Group on Speech and Language Difficulties, 2013; Locke, Ginsborg, & Peers, 2002; Nelson, Welsh, Vance Trup, & Greenberg, 2011). One factor associated with low SES that impacts language development is the richness of the language environment in which children are raised (Hart & Risley, 1995). A number of longitudinal studies have shown that differences in the richness of linguistic input result in an increasing gap in children’s language development (Hoff, 2013; Huttenlocher, Waterfall, Vasilyeva, Vevea, & Hedges, 2010; Reilly et al., 2010; Rowe, Raudenbush, & Goldin-Meadow, 2012), whereas brain imaging evidence has suggested that young children regularly engaged in conversation by adults have stronger structural connectivity between two language regions, Wernicke’s area and Broca’s area (Romeo et al., 2018).

From the point of view of a single mechanism embedded within a wider cognitive system, the deficit in input need not be a property of the external environment, but could stem from deficits in other parts of the system. For instance, one theory of why components of the social–cognitive system (such as those underlying face recognition) do not develop typically in autism is that the infant as a whole does not pay attention to the relevant social cues that are nevertheless present in his or her environment (e.g., Elsabbagh et al., 2011; though see Elsabbagh & Johnson, 2016). Thus a face recognition system might not develop appropriately because it is not exposed to sufficient information about faces.

Behavioral intervention should therefore involve enriching the learning environment from the perspective of the relevant mechanism, to ensure sufficient information is present to acquire the target ability. In the domain of language, there are initiatives to encourage parents from lower SES backgrounds to talk more to their children (e.g., Leffel & Suskind, 2013; Suskind & Suskind, 2015); within autism, interventions are being developed that specifically train infants at familial risk of autism to pay attention to social cues (Wass & Porayska-Pomsta, 2014).

Restoration of an enriched input should bring atypically developing systems back toward the typical range of development. There is one caveat that concerns timing. Certain domains, particularly those involving low-level perceptual skills, may exhibit sensitive periods in development, such that later acquisition does not reach the same ultimate levels of proficiency (Huttenlocher, 2002). Restoration of enriched input that occurs after the plasticity of the system has begun to reduce may not be as successful; in effect, the early disadvantage will be imprinted on the structure of the system. One example of such an account is the proposal that DLD is caused by an early auditory deficit even though not all children with DLD show auditory deficits. The idea is that an early auditory deficit may resolve in some children, but because of sensitive periods in the development of the language system, the now-enriched auditory input cannot bring the development of the language system (and specifically, its phonology) back onto the typical trajectory (Bishop, 1997).

Table 3 shows data from a polygenic model of individual differences (Thomas, 2016a), again employing the example domain of English past tense. Here, development is simulated in 1000 children, with individual differences arising from two sources: variation in multiple computational parameters and variation in the richness of the information present in the learning environment. The population depicted in Table 3 experienced wide variation in the richness of individuals’ learning environments, whereas the variation in computational learning parameters was more restricted, so that environment was the main driver of individual differences (see Thomas, 2016a, for simulation details; GNEW population). Variation in the environment was implemented by a one-time filter on the training set applied to each family, analogous to the effects of SES on language input (Thomas et al., 2013). The top line of each section in Table 3 shows how the population mean and distribution of performance changes across development (in this case, a life span of 1000 epochs of training, where one epoch was a single exposure to the individual’s family training set).[Table-anchor tbl3]

At epoch 50, relatively early in development, every simulated child’s environment was fully enriched to provide the maximum possible training set. Table 3 shows the effect on population means and standard deviations following the onset of intervention. Regular verbs immediately showed an acceleration in response to this whole-population intervention, with variation reducing and the lowest performers eventually performing above the 50th-centile of the original population. Irregular verbs took more time to exhibit the acceleration, indeed initially showing a decline, but eventually exhibited large gains. In general, acquisition of irregular verbs in these associative models tends to be more sensitive to the computational properties of the network. For irregular verbs, variation in computation properties continued to produce consistent individual differences in performance despite the enriched environment and population standard deviation did not change in the developmental phases following enrichment (Table 3, middle section, distributions after 50 epochs). In other words, the gap between simulated children did not close following enrichment. Instead, the whole population increased its performance level. In contrast, gaps *did* close for the easier regular verbs, where computational properties did not constrain performance so strongly; poorer performing children caught up once the hindrance of a disadvantaged environment was lifted. In short, the effects of universal enrichment on narrowing gaps between children depended on the extent to which internal computational properties constrained development.

Functional plasticity can reduce in associative networks with age via a number of mechanisms (Thomas & Johnson, 2006). In connectionist models, age may be indexed by the amount of training the system has experienced or by a maturational schedule acting on computational properties. Among the mechanisms that can reduce plasticity are the loss of resources, reductions in the malleability of connections in response to training signals, entrenchment of connectivity (that is, well established connections take longer to reset), and assimilation (whereby top down processes reduce the detection of differences in an altered learning environment, thereby mitigating the responsiveness of the system to the new conditions).

The population under consideration here experienced aged-related reductions in plasticity through pruning of connectivity, which reduced available resources (or capacity). Pruning had its onset at around 100 epochs. The bottom section of Table 3 shows the effect of population-wide enrichment on irregular verb performance at 250 epochs compared with, respectively, normal (untreated) development and early intervention. Intervention had reduced effectiveness when it commenced after the onset of pruning. For regular verbs, by the end of training, the mean improvement in population accuracy following early enrichment was 22%, whereas that following later enrichment was 16%. For irregular verbs, the improvement following early enrichment was 31% and after later enrichment 13.5% (*t* test, both *p* < .001). Notably, the late intervention *increased* the population standard deviation for irregular verbs: intervention increased the gaps between individuals.

**Figure 3 fig3:**
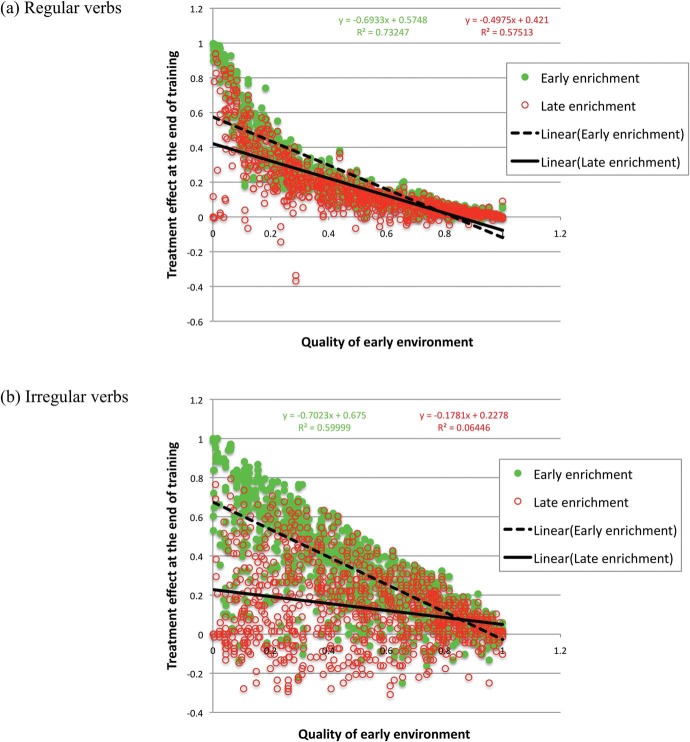
Individual differences in response to an enrichment intervention. Plot shows the relationship between treatment effects (change in proportion correct assessed at end of training) and the quality of the early environment for each simulated child (varying between 0 and 1) for (a) regular and (b) irregular verbs. Poorer family language environment predicted a larger treatment effect. This effect reduced for interventions later in development, and more so for irregular verbs. Early enrichment = 50 epochs, Late = 250 epochs, treatment effects assessed at 1000 epochs. Linear fits are shown for all conditions. Early enrichment for regular verbs was better fit by a log function (*R*^2^ = .87), whereas linear functions explained more variance for the other three conditions.

If early impoverished environments cause deficits, the size of the treatment effect available through enrichment should be inversely proportional to the quality of that early environment. In other words, children who are held back more by an impoverished early environment should have greater scope for improvement following enrichment. In the simulation of early enrichment, this correlation was observed both for regular and irregular verbs, with correlations between environmental quality and treatment effect of −.86 and −.77, respectively (Figure 3a).[Fig-anchor fig3]

However, sensitive periods in development eventually translate the consequence of being raised in a poor environment into a deficit in the structure of the network, which later enrichment is less able to undo. In this scenario, the greater the early impoverishment, the greater the impact on the development of processing structures, and the poorer the predicted treatment effect. One might thus expect the inverse correlation of early environmental quality and treatment effect to weaken or even reverse. In line with this expectation, the equivalent correlations following late enrichment were −.76 and −.25 for regular and irregular verbs, respectively (Figure 3b). The reduction in scope for treatment across development for networks raised in poorer environments was larger for irregular verbs than regular verbs, because they are more sensitive to the processing capacity of the network (in a fully factorial ANCOVA of treatment effects with factors of verb type and timing, and environmental quality as the covariate, all main effects and interactions were highly significant).

The pattern of more sustained early deprivation leading to less easily remediated deficits can be seen in data from a recent follow-up study of Romanian orphans exposed to severe early deprivation but then adopted into enriched environments. Sonuga-Barke et al. (2017) found that, when followed up into young adulthood, Romanian adoptees who experienced less than 6 months in an institution had similarly low levels of symptoms as typically developing controls. By contrast, compared with controls, Romanian adoptees exposed to more than 6 months in an institution had persistently higher rates of symptoms of autism spectrum disorder, disinhibited social engagement, and inattention and overactivity through to young adulthood.

Thus, enrichment interventions to alleviate deficits caused purely by a lack of appropriate experience need to pay attention to possible timing effects impacting plasticity. If plasticity reduces, enrichment alone will be insufficient as an intervention. How should interventions alter if plasticity has reduced? The best behavioral intervention method in the case of late intervention will depend on the particular mechanism causing the plasticity loss for the domain and mechanism in question (see, e.g., McClelland, Thomas, McCandliss, & Fiez, 1999; Thomas & Johnson, 2006). It may involve more intense practice, more feedback, or perceptually exaggerated stimuli. The key message, however, is perhaps an obvious one. Where a theoretical understanding of development in the target domain suggests reductions in plasticity with age in key mechanisms, early interventions to alleviate impoverished experience become more important. If environmental factors (such as SES) inversely predict response to treatment in younger but not older children, this is the hallmark of the operation of sensitive periods.

**Figure 4 fig4:**
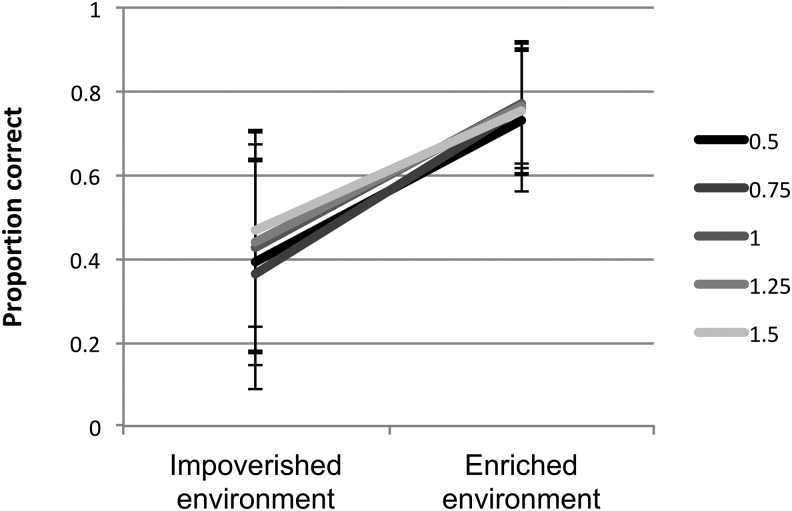
The interaction of processing deficits with richness of early language environment. The plot depicts population performance on regular verbs early in development (50 epochs), split by individuals in impoverished or enriched environments, and stratified by individuals with different unit discriminability (temperature values 0.5–1.5). Interaction effect was at trend level (*p* = .06). Error bars show standard deviations.

Lastly, behavioral deficits produced by impoverished learning environments will not necessarily act independently of differences in intrinsic learning properties. Figure 4 shows the difference between impoverished and enriched learning environments for the simulated population, stratified by their unit discriminability. The effect of learning environment interacted with this internal computational constraint, such that the less optimal computational constraint tended to exaggerate the impact of the impoverished environment, albeit this was a marginal effect against the variation of other computational parameters in the population (main effect of environment: *F*(1, 996) = 89.61, *p* < .001, η_p_^2^ = .083; main effect of temperature: *F*(1, 996) = 10.73, *p* = .001, η_p_^2^ = .011; environment × temperature: *F*(1, 996) = 3.51, *p* = .061, η_p_^2^ = .004). This interaction occurred because both influences act on the strengthening of network connections, which in turn drives behavior. An increase in the incidence of developmental disorders in low SES families may, therefore, represent an interaction between risk factors, rather than resulting from pure environmental effects.[Fig-anchor fig4]

In sum, interventions to remediate deficits stemming from insufficient stimulation of a developing cognitive system may either target the external environment, or the internal environment of the system by seeking to alter those aspects of the external environment to which the child attends. Enrichment interventions will eliminate gaps between children unless the target behaviors are sensitive to other (independently occurring) individual differences in computational properties of learning mechanisms. In the latter case, enrichment can improve the whole population level of performance without narrowing gaps between children. Lastly, environmental effects may interact with and exacerbate underlying computational risk factors.

#### Choosing better training sets to support atypical processing properties

In the first section, we observed how a processing system with atypical computational properties could eventually reach ceiling performance on the training set but show residual deficits in generalization. Supporting generalization is an example where specific additional experience can be used to restructure representations.

Fedor, Best, Masterson, and Thomas (2013) explored how the addition of specially designed input–output mappings could support generalization in networks with atypical processing properties. These authors also employed a feedforward connectionist model drawn from the field of language development, in this case acquisition of the Arabic plural (Forrester & Plunkett, 1994). The aim was to visualize the formation and mediation of atypical representations of categories. The model was trained to learn categorizations defined over a two-dimensional input space using high-dimensional internal representations. Fedor et al. considered different categorization problems, in each case only giving the network a limited sample of the categorization problem, and testing its ability to acquire (generalize to) the full function.

Developmental disorders were then simulated by initial changes to parameters such as the denseness of connectivity, numbers of internal processing units, the learning rate, the unit discriminability, and processing noise. Next, cases of developmental deficits were rerun and interventions applied early in development. Interventions comprised additional input-output mappings (no more than 10% of the size of the training set), which offered different information about the categories. For example, interventions might mark out prototypical members of categories, or demarcate the edges of category boundaries in the input space. The results of these exploratory simulations indicated that the best interventions either sampled the whole problem space or provided a representative ‘slice’ across all categories. There was also some evidence that interventions were differentially effective depending on the problem domain (mapping problem) and depending on the type of deficit.

**Figure 5 fig5:**
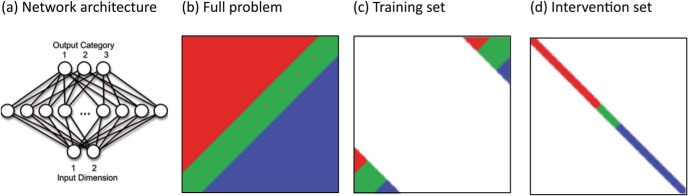
Network architecture and problem domain for a model designed to explore how bespoke intervention sets can support learning in systems with atypical properties, in this case reduced connectivity: (a) network architecture; (b) example categorization problem, with 10,000 data points; the network is required to learn the category boundaries; (c) the training set given to the network, sufficient to learn the problem under typical conditions; (d) an example intervention set added to the training set to aid development under atypical conditions. Networks had 50 internal units (backpropagation network; learning rate = .1, momentum = .3, temperature = 1)

**Figure 6 fig6:**
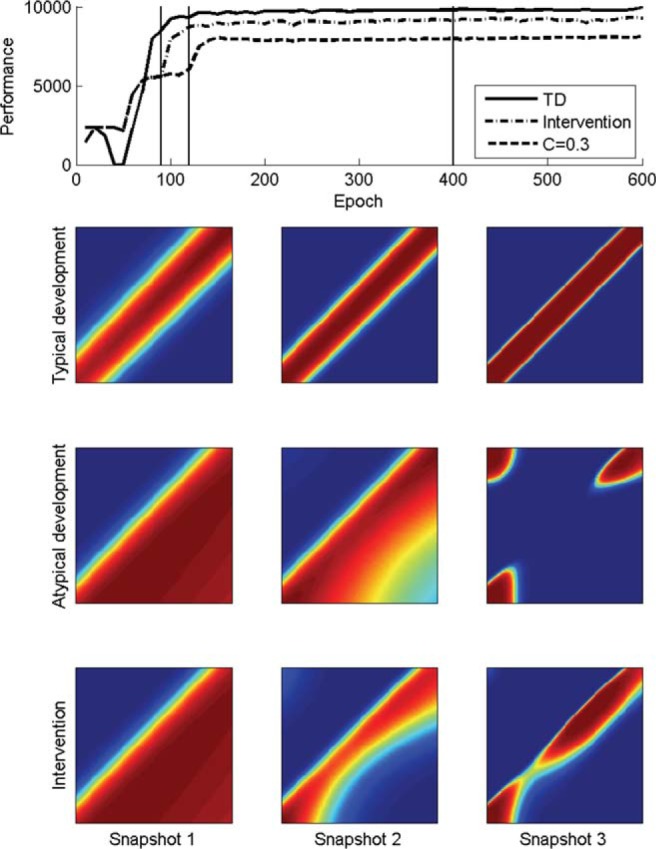
Developmental trajectories and internal representations in a typical case (TD), an atypical case with low connectivity (30%, *C* = 0.3) and the same atypical case experiencing an intervention. Top panel: Developmental trajectories; intervention commenced at 100 epochs. The intervention set was added to the training set for the duration of training. Vertical lines show epochs at which snapshots were taken. Lower panels: snapshots of the activation pattern of the unit for output category 2 in the three cases, which should respond only to the central band (see Figure 6). Hot colors represent more activity. (Fedor et al., 2013).

Figure 5 illustrates one example of a training problem used by Fedor et al. (2013). It shows the architecture, the full categorization problem, the training set (which represents a subset of the full problem), and then an example intervention set. Here, the network had to learn a category that spanned a zone around a diagonal of the two-dimensional input space, with different categories either side. The training set only provided examples at either end of the diagonal, and the network had to learn to *interpolate* the general function linking the two ends. Figure 6 demonstrates an example of a network learning this general function successfully. Although the internal representations of the network had high dimensionality, their structure could be visualized by determining the network’s categorization of all 10,000 possible locations in the input space. Figure 6 shows that in the typical case, there was quick formation of the diagonal category but with fuzzy boundaries, which were then progressively sharpened through further training. The figure also shows the formation of atypical representations in a case of a developmental deficit, in this case, a network with only 30% of the normal level of connectivity. Interpolation was unsuccessful, and eventual performance retained accuracy only in the region of the training set. Finally, the figure demonstrates the consequence of adding an effective intervention (a slice across all categories) early in training. These additional input–output mappings improved performance on the training set, but crucially were also able to support acquisition of the general function despite the atypical processing properties. This is an important demonstration that atypical processing properties may require the design of special intervention sets to support generalization, even in cases where high accuracy on the training set can eventually be reached through extended exposure. Alleviation of the deficit cannot be achieved by more naturalistic experience, but requires bespoke additional training to restructure representations based on a theoretical understanding of the target domain.[Fig-anchor fig5][Fig-anchor fig6]

#### Simplifying the problem the atypical system has to solve

Where a cognitive mechanism is struggling to acquire a target ability, a behavioral intervention might seek to reduce the complexity of the problem the system is trying to solve. It might do so by altering the input and output representations, or restricting training to a subset of the task.

From a computational perspective, a task domain is defined by the set of input–output mappings. The *complexity* of the problem is specified by the way the domain is encoded, with respect to the input representations and the output representations, and the number of mappings to be learnt. Where a learning mechanism has insufficient computational resources to solve the problem, development occurs more slowly, may asymptote at a lower level, show acquisition of some parts of the domain but not others, or show generalization deficits. We have so far considered behavioral intervention as adding some further information to the structured environment or altering its frequency distribution. However, a behavioral intervention could serve to alter the nature of the input or output representations. Changing the representations might simplify the problem that the learning mechanism has to solve, and bring it within what can be achieved with the existing computational constraints. That is, a less powerful mechanism may be able to learn a simpler problem.

Behavioral interventions for dyslexia and word-finding difficulties both appeal to this idea. For reading, some interventions target the structure of the phonological representations, the output of the decoding system. For WFD, interventions additionally target improvements in semantic representations, the drivers of naming. Computational models of intervention have also appealed to this method. Seidenberg and McClelland’s (1989) original connectionist model of reading was later deemed to be closer to the performance of a dyslexic, because it had representations that didn’t show sufficient similarity between written letters or between speech sounds to allow the learning mechanism to generalize the reading problem to novel words. The presentation of the problem domain made it too hard for the learning mechanism to solve. A later implementation utilized more componential input and output representations and was taken to be a better model of typical development (Plaut et al., 1996). One of the interventions considered to alleviate dyslexia in the Harm et al. (2003) model was to improve the output representations developed by the phonological component. Best et al.’s (2015) model considered interventions to improve naming—captured as the mapping between semantic and phonological representations—by treatments that improved the representations of semantics or phonology in isolation, rather than simply more practice in using the compromised pathway linking these representations. Lastly, Harm et al. demonstrated that improvements stemming from changes in input or output representations may be subject to timing effects; previous learning may cause entrenched connections that mean the mechanism responds less readily when representations are changed later in development.

**Figure 7 fig7:**
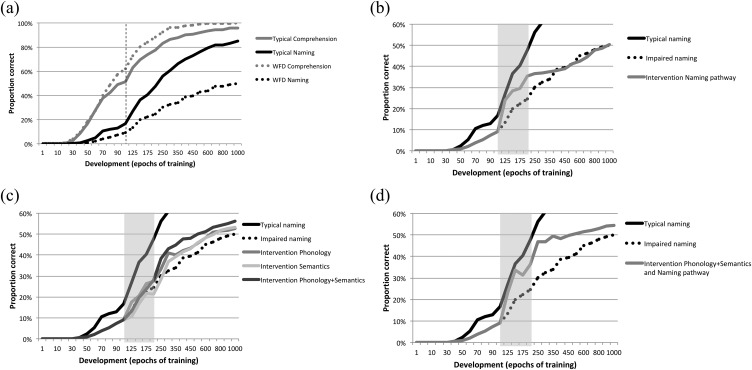
A model comparing interventions to remediate weaknesses or to improve strengths. (a) Developmental trajectories for naming and comprehension in a model acquiring the meanings (semantics) and word names (phonology) of 400 vocabulary items (averaged over 3 replications). The typical model shows the usual comprehension-production asymmetry. In the Word-Finding Difficulty (WFD) model, there was a restriction in the capacity of the pathway linking semantics to phonology (from 175 to 70 hidden units), which impacted on the development of naming, while comprehension trajectories did not reliably differ from normal. (b) Early intervention targeting the naming pathway (weakness). (c) Early intervention targeting the development of the phonological representations, the semantic representations, or both (strengths). (d) An intervention combining training on strengths and weakness. Intervention comprised training at five times the frequency on acquisition of these representations compared with naming and comprehension, beginning at 100 epochs and lasting for 100 epochs, shown by the shaded area. (Alireza et al., 2017).

The Best et al. (2015) model used fairly idealized depictions of semantics and phonology. Figure 7 shows results from a model with more realistic representations (Alireza, Fedor, & Thomas, 2017). Using the same architecture as the Best et al. model, this implementation employed a training set of 400 English words taken from the Masterson, Stuart, Dixon, and Lovejoy (2010) corpus of words found in children’s books. Phonology was encoded in a slot-based scheme using articulatory features, while semantics used a feature-based scheme of more than 1000 features drawn from Vinson and Vigliocco’s (2008) adult ratings of word meanings. Figure 7a depicts the typical model in its development of semantic knowledge, phonological knowledge, single word comprehension, and single word naming; and an atypical network, which had a computational restriction to the naming pathway that linked emerging semantic and phonological representations. For the atypical network, Figure 7b–7d depicts the effect on naming of a relatively short intervention early in training (between 100 and 200 epochs, in a life span of 1000 epochs, depicted by the shaded area). Intervention was triggered at a point when the typical model had acquired a productive vocabulary size of 67 words, whereas the atypical models had a vocabulary size of 36 words. Five different interventions were contrasted, of three types: (a) remediating the weakness—the model was provided with additional training on the naming pathway; (b) improve the strength—the model was provided with additional training to improve the (otherwise typically developing) semantic representations, the phonological representations, or both at once; (c) both Types 1 and 2 were combined into an intervention that sought to simultaneously improve strength and remediate weakness.[Fig-anchor fig7]

The intervention designed to target the naming weakness, extra practice for the semantics-to-phonology pathway, improved performance initially, but served only to propel the system further along its atypical trajectory. The final level of performance was no higher; eventually, the untreated condition caught up with the treated condition. Interventions to target strengths, the semantic and phonological representations, produced more gradual improvements (little during the intervention period itself), but subsequent improvements were long-term and raised the final level of performance. This is because extra training on the input and output representations for naming served to make them more distinguishable, and therefore make the task of learning the arbitrary mappings between meaning and sound easier for the restricted pathway. The largest benefit occurred when both semantic input and phonological output representations were improved (Figure 7c). When the input/output intervention was combined with extra training on the semantics-to-phonology pathway, both short-term and long-term benefits were observed (Figure 7d).

Alireza et al. (2017) also considered the effects of timing, contrasting interventions at 100, 250, and 750 epochs. In all models, unused network connections were pruned away with a small probability from 100 epochs onward, reducing the plasticity of older networks. Later in training, improving strengths became less effective and remediating weaknesses became more effective. Echoing the findings of Harm et al. (2003), the benefit of improving input and output representations was more marked early in development, and reduced once pathways had committed to utilizing the (potentially poor) initial representations. At that point, maximizing the performance of the pathway through intense practice became the best recourse.

In sum, behavioral interventions that improve either the input or output representations involved in acquiring a cognitive domain may improve the ultimate level of performance that is attainable by the system with atypical computational constraints, but such improvements may be subject to timing effects. Remediating weakness did produce improvements, but these only propelled the system more quickly along the same atypical trajectory. In this model, long-term benefits of an early intervention arose from improving strengths, not from focusing on weaknesses. However, the opposite was true of a late intervention.

If input and output representations cannot be altered, how can the problem be simplified to help an atypical mechanism? If the model is unable to learn the training set to a given performance level through limitations in processing capacity, adding further input–output mappings to the training set is unlikely to enhance accuracy on the patterns in the original training set. What one might call normalization through behavioral intervention is therefore difficult if one conceives of developmental deficits as arising from limitations in individual systems. We define *normalization* here as the acquisition of the abilities and knowledge that any typically developing system acquires through exposure to the normal training set.

However, one might take the view that, for adequate functioning of a child in his or her day-to-day environment, learning the full repertoire of behaviors in the target domain is not necessary. Perhaps it is sufficient to learn just some items in the training set, the most frequently required, the most prototypical? This more modest objective might suggest interventions that focus only on a subset of the training set. For example, in the past tense domain, one might select the most frequently used verbs, be they regular or irregular. Alternatively, one might take the view that what the atypical system needs to learn is not the training set per se (even though this is what typical systems acquire), but a general function implicit in the items in the training set. Acquisition of this general function can be assessed by performance on generalization sets rather than the training set. There may then be input–output mappings that can be added to the training set which could improve the network’s ability to extract the general function, even if performance on the original training set did not improve (or even worsened for those parts inconsistent with the general function). In contrast to normalization, we could term this approach *compensation*, because the aim is to optimize a subset of behaviors present in the original training set. In the past tense domain, such an approach might seek to improve acquisition of the regular past tense rule by showing its use across a variety of verb forms.

The distinction between these two intervention aims—improving performance on the full training set versus on a subset or a function implicit in the training set—allows us to draw a formal distinction between normalization and compensation, with respect to our single-mechanism perspective. It poses the challenge of how one might derive interventions that achieve these goals. So far, we have conceived of a behavioral intervention as the addition of training patterns to the network’s training set for some duration. Which additional patterns would support normalization, under our definition? Which additional patterns would support compensation?

Yang and Thomas (2015) explored one method to derive intervention sets within a machine-learning framework. The method assumes the availability of an artificial neural network that is able to successfully acquire the target domain through exposure to the training set. A genetic algorithm technique is then used to identify which input units were most important for generating good learning on, respectively, the training set or the generalization set. Intervention items can be produced which embody the features that support either training set acquisition or generalization. An intervention set then comprises a selection of these items, for example which span the internal representational space of typically developing models. The internal representational space can be characterized by principal component analyses of hidden unit activations produced by the training set. Davis (2017) used this method to derive intervention sets to encourage either normalization or compensation, and applied them to a model of autism. Intervention sets contained around 10% the number of patterns as the training set. The results in that case indicated that compensation was more effective than normalization for networks with compromised connectivity, because in artificial neural networks, regularity is less demanding on representational resources.

The Yang and Thomas method for deriving intervention sets is model dependent. It requires the availability of a fully specified training set, and commitment to the representational format in which the problem is specified. Moreover, compensation requires specification of the implicit function to identify the key input dimensions that embody the function—in other words, a theory of the information that is most important in a domain.

In sum, behavioral interventions may be successful in mechanisms with atypical computational constraints if the goal of intervention is revised from normalization (fully behavioral competency) to a subset of skills, which we termed compensation. Machine-learning methods suggest possible ways of identifying items that will support normalization and compensation.

#### Altering the computational properties of the system

If the atypical computational constraints limiting acquisition of a target cognitive domain cannot be remediated by altering or complementing training experiences, intervention may instead seek to change the computational constraints. Not all theoretical approaches to development view the computational properties of learning mechanisms in the cognitive system as fixed. If computational properties can be influenced by experience, this opens up the possibility that behavioral intervention could alleviate computational limitations and enable successful remediation. In its development, the brain undergoes a phase of elaboration of connectivity followed by regressive events that prune away connectivity; in addition, some existing connectivity is enhanced by myelination (Huttenlocher, 2002). It is as yet unclear what direct bearing such brain-level changes have on cognitive development. Researchers have sometimes included both increases in connectivity and decreases in connectivity in their developmental cognitive models. For example, constructivist approaches employ networks that can increase the number of processing units and connections in an experience-dependent manner (see, e.g., Quartz & Sejnowski, 1997; Mareschal & Shultz, 1999; Westermann & Ruh, 2012). Other models have included pruning of connectivity, where the connections removed are those that have not been strengthened by experience (e.g., Thomas, 2016a). Yet other models have included the assumption that some computational properties alter according to a maturational schedule. For example, Munakata (1998) captured age-related differences in a connectionist model of the infant A-not-B task partly through a maturational increase in the system’s ability to maintain active representations, implemented by a gradual increase in the strength of recurrent connections.

In principle, then, one could conceive of a behavioral intervention modulating a mechanism’s computational properties through altering the way certain parameters change across development. For example, this might equate to stimulation causing greater elaboration of connectivity in the target mechanism, or greater resistance to loss of connectivity during pruning of connectivity. To illustrate how this might work, consider a model of autism proposed by Thomas et al. (2011). This account initially focused on the regressive subtype. It proposed that autism is caused by an exaggeration of the normal phase of pruning of connectivity occurring from infancy onward; overpruning occurs and particularly impacts long-range connectivity. Thomas, Davis, et al. (2016) later showed how differences in the timing of onset of overpruning could link early onset, late onset, and regressive subtypes of autism (Landa, Gross, Stuart, & Faherty, 2013). Davis (2017) then considered whether the behavioral deficits shown by the atypical connectionist models could be remediated by interventions of different types and applied at different times. Behavioral improvements were on the whole relatively small, and individual networks show variation in their response to intervention. However, some networks did show a marked behavioral benefit from a short, discrete intervention applied early in development.

**Figure 8 fig8:**
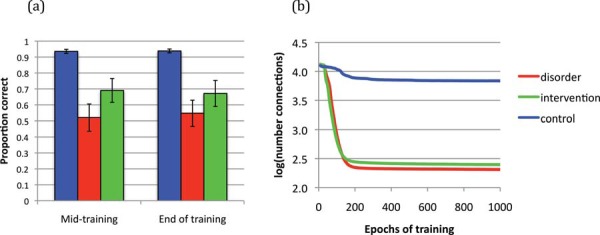
A behavioral intervention to alter computational properties, in this case, to protect against overpruning of connectivity. (a) Performance of a group of 9 networks with a disorder caused by greater-than-usual loss of connectivity (red [dark gray]), compared with control networks (blue [middle gray]). Also shown are the disorder networks following an early behavioral intervention (green [light gray]), lasting between epochs 30 and 70. Effects of the intervention sustain to the end of development. (b) The number of network connections for the disorder group in untreated and intervention conditions. The intervention caused initial acceleration of loss but final preservation of a greater proportion of connections, associated with improved computational power. Midtraining = 250 epochs; End of training = 1000 epochs.

Figure 8 shows the mean performance of a group of such networks that exhibited a strong response to early intervention. Networks were trained for 1000 epochs, with the onset of pruning between 25 and 50 epochs; atypical networks were exposed to an intervention at epoch 30, lasting 40 epochs; the intervention was designed to enhance generalization by including novel examples of items following the implicit rule present in the training set, with the intervention set approximately 10% the size of the training set. Figure 8a shows the behavioral deficit of the impaired networks, compared with a control condition of the same networks trained without the atypical setting of the pruning parameter. The short intervention showed a marked benefit on accuracy, which sustained until the end of training. The size of the intervention effect was highest in midtraining, and did not increase at the later measurement point. Figure 8b shows the total number of connections in the atypical networks in the untreated and treated conditions. Notably, during the intervention, connection loss accelerated as the internal representations underwent reorganization. Thereafter, the treated condition retained a greater proportion of connections (*t* test: 250 epochs *t*[8] = 3.91, *p* = .004, Cohen’s *d* = .43; 1000 epochs, *t*[8] = 3.85, *p* = .005, *d* = .37). Connection number is associated with improved computational power.^3^ The behavioral intervention for these atypical networks, then, served to improve their computational properties during subsequent development compared with the untreated condition. Here, the stimulation of the intervention produced greater resistance to loss of connectivity.[Fig-anchor fig8]

Under a maturational view, computational properties may alter with development, but the schedule is not influenced by behavioral interventions, or more broadly, by experience. (Under such an account, it is not that the experience plays no role in development; it is just that experience is not the limiting factor on rates of growth.) In such a scenario, behavioral interventions could be rendered successful by waiting until the computational properties have improved. Maturational accounts have been proposed in disorders such as DLD (Bishop & McArthur, 2004) and ADHD (Batty et al., 2010; Shaw et al., 2007). Evidence from neuroscience has been used to argue that interventions for anxiety disorders may be more effective after adolescence due to the developmental state of the underlying mechanisms (Hartley & Casey, 2013). Within the field of education, the broader notion of school readiness is predicated on the assumption that development of skills such as executive function needs to have reached a certain level before the classroom-based behavioral methods can be properly effective (Noble, Tottenham, & Casey, 2005).

A further alternative would be to directly manipulate the computational properties of the processing mechanism. We refer to these as *biological* interventions, because they need not involve behavioral methods directly relevant to the target skill. Biological interventions most obviously would include pharmacological treatments that alter the levels of neurotransmitters (e.g., dopamine for ADHD, Volkow, Fowler, Wang, Ding, & Gatley, 2002; serotonin for repetitive behaviors in pervasive developmental disorders, McDougle, Kresch, & Posey, 2000; oxytocin in autism, Preckel, Kanske, Singer, Paulus, & Krach, 2016). More speculatively, biological methods might target neural activity via electrical methods (e.g., direct cortical stimulation for dyscalculia; Iuculano & Cohen Kadosh, 2014) or brain plasticity via drug treatments (e.g., valproate acid for auditory learning; Gervain et al., 2013). Biological methods might also employ behavioral practices that do not directly target cognition but influence brain function, such as exercise and diet (e.g., for treating ADHD: alterations of diet, Konikowska, Regulska-Ilow, & Rǒzańska, 2012; use of exercise, Silva et al., 2015). Or they might employ methods that indirectly target cognition, for example through the effect of sleep on memory consolidation, or mindfulness training on attention, or action video game playing on visual attention (e.g., role of sleep in developmental disabilities: Ashworth, Hill, Karmiloff-Smith, & Dimitriou, 2017; Dodge & Wilson, 2001; mindfulness treatments for autism, dyslexia, ADHD: Sequeira & Ahmed, 2012; Tarrasch, Berman, & Friedmann, 2016; video game playing for dyslexia: Franceschini et al., 2013).

It should be possible to construe all such biological effects in terms of manipulations to parameters within computational models of development. For example, impulsivity in ADHD has been modeled in terms of a computational constraint on reward-based or reinforcement learning. Williams and Dayan (2004, 2005; Richardson & Thomas, 2006) used one form of reinforcement learning, Temporal Difference learning, to simulate a developmental profile of impulsivity in ADHD, based on a model of the role of dopamine in operant conditioning. In this model, the agent (child) had to learn to delay an immediate action that gained a small reward in favor of a later action that gained a larger reward. Williams and Dayan simulated ADHD by altering the ‘discounting rate’ parameter, which determined the relative weighting of immediate versus long-term rewards in guiding action. The atypical setting of the parameter corresponded to the lower levels of dopamine found in the brains of children with ADHD. A system that discounted long-term rewards developed impulsive behavioral patterns, by allowing small immediate rewards to guide action. Although this model was not extended to consider intervention, the common pharmacological treatment for ADHD, methylphenidate hydrochloride, is a stimulant that operates by increasing levels of dopamine in children’s brains (Gottlieb, 2001). In the model, the effects of the biological intervention could be simulated by altering the discounting rate parameter, thereby removing the atypical constraint on subsequent development of impulse control in reward-based action decision-making.

Harm et al.’s (2003) reading model in effect included a biological intervention. In one of its conditions, an initial computational limitation in the phonological component (lower connectivity and restrictions on weight size) was simply eliminated by an intervention. Lost connections were restored and weights were allowed to take on larger sizes. It is worth noting that in this model, this biological intervention was subject to timing effects. Later interventions were less effective because they could not reverse entrenched weight values produced by earlier learning in the network connecting orthographic inputs to atypical phonological outputs. On the face of it, biological interventions might seem more powerful, but they too may be subject to limitations.

### Interventions to Encourage Compensatory Responses Through Other Pathways and Mechanisms

We have thus far construed intervention as targeting the mechanism exhibiting the developmental deficit. However, behavioral interventions might seek instead to encourage the recruitment of other mechanisms or pathways able to deliver or support the target behavior. Models of deficits frequently make reference to pathways outside of the single implemented mechanism to explain behavioral patterns. For example, in Abel, Huber, and Dell’s (2009) model of acquired naming deficits, the authors referred to a range of additional structures not realized in their implementation as possible sources of naming errors. These included visual input, the conceptual-semantic system, an editor component, and a phonetic component. When Plaut (1996)’s model of acquired deep dyslexia was unable to accommodate a certain pattern of reading errors during relearning after damage, Plaut argued that the pattern originated from the operation of an unimplemented phonological route. In their model of developmental dyslexia, Harm et al. (2003) argued that interventions acting on an unimplemented semantic route would improve word reading rather than just the nonword reading improvements shown by the implemented architecture.

Some disorders may even originate from atypical organization of pathways, rather than limitations in particular mechanisms. For example, Chang’s (2002) connectionist model of sentence production demonstrated how inappropriate sharing of information between mechanisms (in this case, those responsible for processing sequencing information and message information) caused a marked developmental impairment in generalization (Dell & Chang, 2013). The model learned to produce sentences in the training set, but was poor at generalizing words to appear in functional roles it had not encountered. In a similar way to Thomas’s (2005) model of compensated morphosyntax in DLD, this model had acquired an overly lexicalized approach to acquiring syntax. More generally, lack of separation of information can in some cases make the computational task much harder for a system to solve (see, e.g., Norris, 1991; Richardson & Thomas, 2006). Disorders may also arise when the balance between different inputs driving a mechanism is disrupted. Amblyopia is a well known and much researched disorder of vision where the input from one eye is weaker than the other; one eye comes to dominate processing at a cortical level, to the disruption of binocular vision (Thompson, Chung, Kiorpes, Ledgeway, & McGraw, 2015; see Crewther & Crewther, 2015 for a neurocomputational account).

Evidence from functional brain imaging of developmental disorders has encouraged the view that in some cases of good developmental outcomes, usually following intensive interventions, compensatory mechanisms have been engaged beyond normal circuitry, thereby exploiting alternative pathways. For example, arguments have been made in the case of dyslexia (compensatory activation in right inferior frontal gyrus; Hoeft et al., 2011) and autism (compensatory activations in several left- and right-lateralized regions identified in a language comprehension task; Eigsti et al., 2016). Researchers hope that identification of these alternative brain pathways can be translated into new interventions that will encourage adoption of compensatory strategies.^4^

Similar claims for compensatory outcomes have been made on behavioral evidence alone. For example, De Haan (2001) pointed out that in children with autism, despite evidence that individuals processed faces atypically (such as the unusual absence of categorical perception of facial expressions), some nevertheless performed in the normal range on expression-recognition tasks. These individuals tended to have higher IQs. De Haan argued that there must be “a degree of plasticity in the developing system that allows for development of alternative strategies/mechanisms in face processing” (2001, p. 393).

The proposal that alternative combinations of mechanisms can deliver similar behaviors, which underpins hopes of compensatory outcomes, requires a certain kind of developmental theory to be true—that there is a suite of cognitive mechanisms with differential properties, and development partly involves selecting a combination that will deliver behavioral mastery. In this way, Price and Friston (2002) have argued for *degeneracy* in the brain’s realization of cognition. Degeneracy is a biological concept, whereby elements that are structurally different can perform the same function or yield the same output. For example, objects can be recognized either on the basis of their global shape or by the presence of distinguishing features. The different cognitive functions of either global form or local feature processing can therefore deliver the same output: accurate object recognition. How well a processing component performs a task then depends on the fit of its structure (i.e., its neurocomputational properties) to the intended function; and how much training the component has had in performing the task. Even within the normal range, individuals may follow developmental trajectories that harness different combinations of components to perform the same task. Degeneracy may therefore explain both individual variation in functional brain activations, and variation in impairments following the same localized brain damage (Price & Friston, 2002).

However, relatively few computational accounts have explicitly considered how development could integrate multiple mechanisms to perform complex tasks, let alone how variation in outcomes could arise between individuals. In the mixture-of-experts approach (Jacobs, 1997, 1999; Jacobs, Jordan, Nowlan, & Hinton, 1991), the initial architecture is comprised of components that have different computational properties. A specific mechanism gates the contribution of these components to the output. When the overall architecture is presented with a task, the gating mechanism mediates a competition between the set of components, allowing the most successful component for each training pattern both to drive output performance and to update its weights to become better at that pattern. Across training, certain mechanisms come to specialize on sets of patterns, by virtue of having an initial (perhaps small) advantage in processing those patterns. Why might such a process of emergent specialization differ between individuals? Presumably, variation in outcomes could arise from differences in the set of experts, differences in the experts’ respective computational properties, the operation of the gating mechanism, and the composition of the training set (see Thomas & Richardson, 2005).

As yet, no computational accounts have considered how an intervention might alter the organization of a set of mechanisms to improve accuracy on a given behavior - for our purposes, directing learning toward mechanisms with fewer restrictions on their plasticity. We do know that in practice, clinicians tend to shift from implicit to explicit methods with older children, to encourage compensatory strategies, suggesting that metacognition might be efficacious in triggering a reorganization of mechanisms. However, there is a missing link in the argument. Although there is evidence of individual variability in the use of mechanisms, and evidence of compensatory engagement of new mechanisms in some disorders where individuals show good outcomes, this does not guarantee that we can generate interventions to encourage the use of alternative sets of mechanisms. That is, evidence of different outcomes *across* individuals is not the same as evidence that all outcomes are equally accessible *to a single individual*. One view is that individual variability in the use of different mechanisms for a task indexes the scope for compensatory reorganization (e.g., in the domain of reading: Kherif, Josse, Seghier, & Price, 2009; Richardson, Seghier, Leff, Thomas, & Price, 2011; Seghier, Lee, Schofield, Ellis, & Price, 2008). But evidence from the functional imaging of compensated brains minimally requires translation to the cognitive level to understand what the compensations represent, before a facilitatory intervention can be developed.

How might an intervention prompt use of compensatory mechanisms? Perhaps a behavioral method could emphasize different task-relevant information, or different modalities; or encourage differential reliance on motor versus sensory demands of the task; or engagement of different representational formats, such as gesture to support language, or language to support spatial cognition. Perhaps atypical overconnectivity could be discouraged by presenting materials that carried less information and therefore engaged fewer mechanisms; disorders of disrupted competition could be remediated by blocking the stronger pathway to allow the weaker to develop, as in the case of patching the stronger eye in amblyopia. This remains to be clarified. Thus, although intrinsic computational limitations in a target mechanism might be overcome by recruiting other mechanisms able to support task performance, or altering the competition and cross-talk between mechanisms, a computational analysis of this strategy is not far advanced, nor an understanding of how to encourage such recruitment via a specific behavioral intervention.

### Individual Differences in Response to Intervention

One of the most challenging aspects of intervention is the variation in children’s response to the same intervention, and the consequent requirement that intervention be tailored to the individual child. How can the therapist determine which intervention is the best to pursue for a given child?

**Figure 9:**
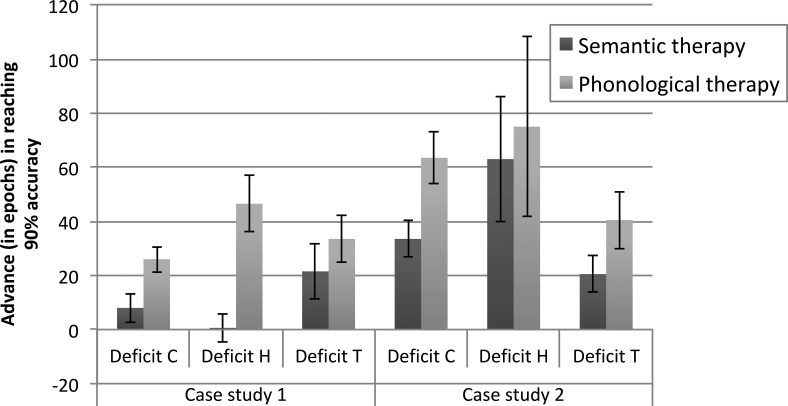
Different computational deficits producing the same behavioral impairment respond differently to intervention. Data show treatment effects of phonological versus semantic interventions for the Best et al. (2015) model of word-finding difficulties, where equivalent behavioral impairments were caused by three different underlying computational deficits. The atypical language profiles of two individual children were simulated and then interventions applied (here measured in how much naming development was advanced). The profile of each child was simulated either by reduced network connectivity (Deficit C), reduced hidden units (Deficit H), or a shallower sigmoid activation function in the artificial neurons (Deficit T). Intervention responses differed depending on how the deficit was implemented. Error bars show standard errors of 10 replications of each intervention (See Best et al., 2015, for further details).

Monogenic models of disorders give some basis to consider differential responses to intervention. For example, in their model of word finding difficulties, Best et al. (2015) were able to use three different atypical constraints (operating on hidden units, connectivity, and unit activation function) to simulate the language profiles of individual children. Figure 9 shows the response to two different interventions (semantic therapy, phonological therapy) for the three different versions of each child with WFD. Notably, the different computational deficits to produce the *same* atypical behavioral profile were associated with different responses to intervention. As with Thomas and Knowland’s (2014) model that sought markers to predict resolution or persistence of delay, the implication here is that measures of underlying processing are necessary to complement behavioral profiles. Indeed, using the enrichment intervention (see Figure 3) but now applied to the Thomas and Knowland (2014) model, networks whose delay would have resolved anyway were found to respond better to intervention than those whose delay would persist. In these associative models, therefore, untreated outcomes are linked to individual differences in response to intervention.^5^

**Figure 10 fig10:**
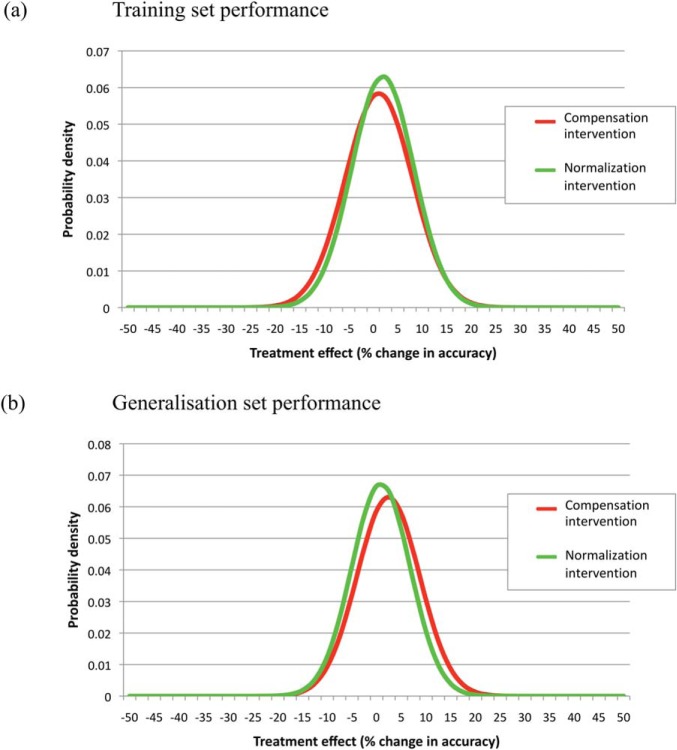
Individual differences in response to intervention, following two types of intervention. Developmental deficits were caused by an overpruning disorder (Davis, 2017). The *x* axis shows treatment effect in terms of change in proportion correct. (a) Distribution for performance on the training set following the *normalization* or *compensation* treatment; (b) distribution for performance on the generalization set following either *normalization* or *compensation* treatment. [Population of 1000 networks, intervention for duration of 40 epochs applied early in development, epoch 30 of a life span of 1000, performance tested at 100 epochs].

Polygenic models of disorders offer a more ready framework to capture differential response. Using population-level models, atypical computational constraints can be simulated against a background of small population-wide variations in many computational constraints, such as those involved in specifying the network architecture, processing dynamics, and plasticity, as well as differences in environmental stimulation. One might think of this as the general intelligence of a network. Figure 10 shows distributions of treatment effects from the simulations of Davis (2017) for a model of regressive autism. The developmental deficit was caused by a single atypical parameter affecting connection pruning, against the background of typical population-wide variation in all other computational parameters. Results were considered separately for training set performance or generalization performance, and in response to normalization or compensation interventions. The treatment effects were generally small, of the order of a few percentage points of accuracy against deficits of 20% to 40%; however, they varied widely across individual networks, including cases of large gains and large losses in response to intervention. Davis (2017) was then able to explore the parameter sets of individual networks to predict the size of the treatment effect, to construct a mechanistic account of the origin of variable response to intervention.[Fig-anchor fig10]

Table 4 shows a set of standardized coefficients from linear regressions for each intervention type, assessed on training set and generalization. The shaded rows represent parameters related to the pathological process (overpruning), the rest to general intelligence. Several points are notable. First, the main effects of these parameters explained the minority of the variance in response to intervention. Although there was a stochastic element to the response, replication indicated that the test–retest correlation was around 0.5, indicating that a fair proportion of the response to intervention depended on the network’s developmental conditions (its parameters and its environment). Mostly likely those development conditions arose from higher order interactions between computational parameters, enabling some networks to gain from intervention, others not to gain, and some to lose. Second, some predictors of individual response depended on intervention type (normalization vs. compensation). Third, predictors could be differentially important for intervention responses on the training set versus generalization, that is, dependent on the target behavior. And last, although some predictors were involved in modulating the impact of the atypical connectivity pruning process, others represented parameters *unrelated to the pathology*, consistent with the idea that general individual differences factors influence the effectiveness of behavioral intervention.[Table-anchor tbl4]

The narrow focus on individual cognitive mechanisms feels particularly restricting in the context of individual differences, where the intervention situation is influenced by many qualities of the whole child, including their attention skills, personality, motivation, and engagement with the therapist in a productive social interaction (or depending on delivery mode, with a teaching assistant, teacher, parent, group of children, or computer). From the single-mechanism perspective, we are restricted to viewing these as factors potentially influencing the plasticity of the mechanism, the information experienced by the child in the therapeutic situation, and the effective dose delivered by the intervention. The child’s attention/motivation/engagement in the therapeutic situation is a necessary precondition for the intervention to gain access to and alter the functioning of the target mechanism. This is somewhat unsatisfying, but is a necessary simplifying step in trying to build a mechanistic account of the sources of individual variability in response to intervention.

## Discussion

We set out to investigate the potential of connectionist modeling to increase understanding of the mechanisms underlying interventions in developmental disorders. We presented and analyzed a range of models and results. To evaluate the potential, let us set a sceptical bar that needs to be cleared. On the one hand, one could have reservations about the use of computational models to simulate development and individual differences by arguing that the models are *too complex*. Connectionist models have many components and components can vary along multiple dimensions (e.g., component: hidden units; dimensions: number of layers, units per layer, pattern of connectivity, activation function). Components and their dimensions are not independent, and behavior results from complex interactions among them (Thomas et al., 2016). These interactions can be difficult to analyze, making it hard to derive deeper principles or generalizations. Perhaps then, the models are too complicated to be useful; and the challenge of mapping from the specific properties of the model to properties of people too great. On the other hand, one could have reservations that the computational models are *not complex enough*. We focused mostly on individual cognitive mechanisms or limited numbers of pathways. The actual cognitive system is far more complicated; we did not consider sensorimotor components, emotional components, social components, executive function components, metacognition, and motivation, let alone the dynamics of the therapeutic situation that we outlined in the introduction. The computational analysis demonstrates that high-level behaviors, and developmental deficits in these behaviors, are determined by complex, nonobvious interactions among multiple factors, some of which cannot be directly measured. Moreover, the modeling suggested that similar looking behavioral deficits can arise from different underlying causes, which in turn respond differently to intervention. Perhaps the sensible conclusion would be that to intervene, rather than investigating underlying mechanisms, it would be better to focus on the behaviors in question and improve them by whatever methods seem effective. Do the findings clear this bar?

### Main Findings

A cognitive mechanism exhibiting a developmental deficit in the behavior to which it contributes does so because exposure to naturalistic experience or to typical educational experiences has not been sufficient to acquire age-appropriate skills. Simply driving this mechanism harder with more experience may not remediate the deficit, just serve to propel it further along an atypical trajectory. This perhaps chimes with the general difficulty of treating developmental disorders, particularly those with pervasive effects such as autism (Charman, 2014a). How can an intervention succeed where naturalistic experience has not?

The simulations we described pursued four lines of investigation. First, we considered *long-term outcomes in the absence of intervention*, exploiting the opportunity of a model, matched to an atypical profile early in development, to project forward to the adult state. Results indicated that processing mechanisms could reach compensated outcomes with expertise in skills less sensitive to the atypical processing constraints but residual deficits in other areas. Resolution in early delays occurred where the cause of the initial deficit was a limitation in plasticity, rather than capacity. Plasticity could be operationalized in terms of a child performance on learning tasks, whereas capacity could be operationalized as the quantity of information that the child can integrate online. Resolution might be accelerated by a greater dosage of otherwise naturalistic experience (i.e., practice). However, early behavioral profiles were poor predictors of these differential outcomes, and measures of processing were needed to improve predictive power (e.g., Fernald & Marchman, 2012).

In the second line of investigation, we considered *methods to remediate atypical development in a single network*. These models addressed, respectively, remediating disorders arising from a lack of early stimulation, choosing a better training set to support atypical processing properties, improving input and output representations, and altering the computational properties of the system. If the deficit in fact arises through insufficient stimulation of the target mechanism, whether externally in richness of the environment to which the child is exposed or internally in the information provided to the single mechanism (for instance, by attentional orienting systems), then the deficit can be treated by alleviating this shortfall. This might amount to enriching the environment (e.g., in the domain of language, with more child-directed speech; e.g., Suskind & Suskind, 2015); or to training attentional mechanisms (e.g., in the case of young children with autism, training attention to social cues, e.g., Powell, Wass, Erichsen, & Leekam, 2016; Wass & Porayska-Pomsta, 2014).

Several possibilities arose for accommodating the atypical processing constraints of the target mechanism: of supporting generalization by additional training on experiences that highlight the structure of the problem domain; of using intervention to alter the quality of the mechanism’s input and/or output representations, thereby simplifying the computational problem that the target mechanism is required to solve; and of training the target mechanism not on the full cognitive domain but a subset of the problem adequate for everyday functioning. Then there were methods that might alter the atypical computational constraints themselves, perhaps in systems where stimulation can cause a change in computational properties; or through delaying intervention in systems where computational properties mature; or using biological interventions to directly alter computational properties (e.g., through pharmacological treatments, or behavioral techniques such as changes in diet, exercise, mindfulness training, action video game playing, and sleep regimes).

In the third line, we considered *interventions to encourage compensation via alternative pathways or mechanisms* to produce the same or similar behavior. Here, computational analysis is less far advanced, mainly because typical models of development have not articulated how a complex system with a suite of cognitive mechanisms can recruit and integrate the mechanisms for behavioral mastery. It is therefore not clear how an intervention could alter the organization of mechanisms to improve task performance. The fact that clinicians shift from implicit to explicit methods with older children to encourage compensatory strategies suggests that metacognition might be efficacious in triggering a reorganization of mechanisms. Metacognitive processes are rarely implemented in models (though see Hoffman et al., 2018, for a recent model of semantics that includes mechanisms to control retrieval). We take metacognition to act by altering internal feedback to the target mechanism, using executive functions to activate or inhibit different pathways and mechanisms, or altering attention to dimensions of the stimulus or required response. Future models that capture such processes are required for a firmer foundation to explore interventions that prompt reorganization.

In the fourth line of investigation, we considered *individual differences in response to intervention*. More recent polygenic models of developmental disorders were useful here, because they simulated the atypical mechanism against a background of typical variation in a range of developmental factors, or indeed captured the developmental deficit as lying on a continuum of population-wide variation (Thomas, Davis et al., 2016). A model investigating the causes of language delay (Thomas & Knowland, 2014) pointed to the limited power of early behavioral markers in predicting whether delays would resolve, because early profiles are largely conditioned by the structure of the task domain. The model suggested that predictive power could be increased by measures of underlying cognitive processes (see Fernald & Marchman, 2012). Notably, the computational properties in the model that led to resolution of early delay also increased responsiveness to intervention. A model investigating individual differences in response to intervention (Davis, 2017) demonstrated that responses could be highly variable, and that both differences in the severity of atypical computational constraints and in other population-wide individual differences factors predicted the response. However, there were stochastic factors, and the predictive factors themselves showed strong interactions such that much variance in outcome remained unexplained, despite replicable individual differences in response to intervention. Finally, a lower level of stimulation from the environment could also play a role, exaggerating the effect of atypical computational constraints (see Figure 4), or itself causing deficits in combination with maturational changes in network connectivity (see Figure 3). Overall, this avenue of modeling is important to support the search for stratification biomarkers in research on developmental disorders, work which seeks to isolate measures (e.g., age, gender, intellectual ability, comorbidity of deficits) that predict developmental outcomes and response to intervention.

Computational insights need to be translated to actual interventions. How might the findings translate into clinical advice? Generalization might be enhanced by an intervention that highlights key cues, or in compositional domains, component parts of stimuli, which would normally be extracted by a typically developing system but need to be included in the experience of a system with atypical properties. If a behavior requires learning associations between representations in different domains, improving these representations may aid an intervention targeting the associations themselves. If there is domain evidence supporting maturation in the target mechanism, waiting to apply the intervention may yield benefits, since computational limitations may reduce with time. Note that this is at odds with the general rubric of intervening earlier at a time of purportedly highly plasticity, but it requires a specific evidence base of the importance of maturation for a given process (see, e.g., Karmiloff-Smith, Casey, Massand, Tomalski, & Thomas, 2014, for discussion of the efficacy of CBT to treat anxiety disorders at different ages, depending on the maturation of fear extinction mechanisms). For older children, explicit interventions may increase the opportunity to engage alternative mechanisms and pathways to drive the impaired behavior, to the extent that metacognition is efficacious in enlisting them. An analysis of the cognitive domain may indicate subsets of behavior that could provide adaptive functioning in everyday life, and so form a compensatory intervention. Lastly, where behavior does not improve through behavioral means, then opportunities can be explored for interventions that alter the computational properties by biological or indirect behavioral means.

#### General principles of intervention

The review of computational work indicates several important factors in the mechanisms underlying intervention effects. First, *the nature of the computational deficit matters*. Similar behavioral deficits can be produced by different underlying computational deficits—all characterized by slower development—but which then respond differently to intervention. Some computational deficits will allow eventual resolution of behavioral deficits, with more experience required to deliver the same amount of behavioral change (such as a reduced learning rate). Some computational deficits will allow partial resolution of behavioral deficits, altering the kinds of abilities that can be supported by the mechanism (such as a less discriminating activation function). Some computational deficits permit eventual good solutions with adapted training regimes (reduced connectivity, supported by a wider range of training examples). Other computational deficits will restrict the ultimate level of behavior that can be supported by the mechanism, leading to persisting deficits (such as fewer hidden units).

Second, *timing matters*. Age was represented in two ways in the models we considered. It could be indexed by an accumulation of previous experience. The Harm et al. (2003) reading model demonstrated a negative effect of prior learning on the potential for intervention, to explain why oral language interventions would have limited success in alleviating difficulties once the child had started to read. Even if the oral language intervention alleviates a core problem in phonology, it cannot undo prior learning linking orthography to atypical phonology. These suboptimal mappings must be overwritten by a complementary intervention targeting decoding. Alireza et al. (2017) found a similar effect in their model of word-finding difficulties. Later in the model’s development, improving strengths (the input and output representations) became less effective and remediating weaknesses became more effective. Once pathways had committed to utilizing the (potentially poor) initial representations, maximizing the performance of the impaired pathway through intense practice became the best recourse. Age could also index maturational changes in the computational properties of the learning mechanism. In the model simulating the effects of insufficient stimulation, late interventions were less successful because maturational pruning of connectivity had consolidated an environmental disadvantage into a structural deficit. Researchers have speculated about the cognitive domains in which maturational constraints may have most impact on training effects (Jolles & Crone, 2012). Sensitive periods suggest early intervention is better, but these reducing profiles of plasticity tend to be limited to lower level sensory and motor domains, rather than high-level cognitive abilities (Huttenlocher, 2002). In some domains, such as attention, training may indeed be more effective in later childhood—at younger ages, the target systems may be computationally immature (e.g., at 4 years instead of 6 years for attention training; Rueda, Rothbart, McCandliss, Saccomanno, & Posner, 2005). A life span perspective suggests that while behavior is changeable at all ages, behavioral changes rely on the brain systems that are most plastic at the age when training takes place (Bengtsson et al., 2005).

Third, *the content of the intervention matters*. We drew a distinction between additional practice on items in the child’s natural experience of the domain and the introduction of new items that highlight key information for the child, such as indicating compositional structure. We additionally distinguished information intended to support generalization of implicit regularities of the cognitive domain to new situations. We distinguished tasks that directly target a behavior compared with those that enhance representations that drive the behavior. We emphasized principles derived from statistical learning theory as candidates to improve learning: the richness of learning experiences, their variability, the provision of novelty in familiar contexts, and the construction of more complex representations from simpler ones. These principles were caveated by the possibility that what works in a system with typical computational learning constraints may not have the same effect in systems with atypical constraints. Lastly, implementation encouraged a focus on the dosage, duration, and regime of training. In distributed connectionist models, modification of the training set can cause interference with prior established knowledge (so-called catastrophic interference; McCloskey & Cohen, 1989; Ratcliff, 1990). Interference can be reduced by lowering the dosage of new information, extending its duration, and interleaving it with training on the old information.

Two important issues in interventions concern *persistence* of interventions effects, and *generalization* beyond items in the intervention set. Beginning with persistence, in a review of persistence and fadeout in the impacts of child and adolescent interventions, Bailey et al. (2017) argued that impacts are likely to persist either for interventions that build skills influencing future development (especially that allow the individual to stay on track in home, school, or community), or in the case of environments that sustain the gains. Skills most likely to yield long-term impact are those that are fundamental for success, malleable through intervention, and that would not develop eventually in the absence of the intervention. The simulations we considered either implemented intervention as an alteration to the training set for a discrete period, or as a permanent alteration. The latter could be viewed as the provision of a sustaining environment for the intervention (such as training parents to permanently altering their interactions with the child, perhaps in their level of language input). Simulation results pointed to persisting benefits of the intervention if the change to the training set was permanent. Discrete interventions could have persisting benefits, but only when plasticity was reduced during training (Davis, 2017), not when it was constant across training. When plasticity was constant, Yang and Thomas (2015) found that early interventions showed dissipating effects across development once the intervention was discontinued, with the exact type of intervention becoming less relevant. In these models, therefore, early discrete interventions had long-term benefits if the consequent gains were consolidated in the structure of the target mechanism. This reveals the double-edged sword of plasticity: if plasticity is consistent across age, interventions can be applied at any age, but the effects of early discrete interventions will be lost; if plasticity reduces with age, interventions must be early, but their effects will persist.

Turning to generalization, because most of the models considered here focused on individual mechanisms, there was not scope to consider the wider issue of far transfer/generalization of training effects to different skills. Nevertheless, when simulating interventions, at no time did we consider improvement on the intervention items themselves—in a sense, this would be trivial, because in error-correction networks, performance on the intervention items will almost always improve. We instead considered transfer from the intervention set to items either in the network’s usual experience (the training set) or to previously unencountered items (the generalization set). This might explain the relatively small size of the intervention effects in a number of cases (e.g., see Figure 10). Results also pointed to the importance of the composition of the intervention set in supporting performance on the training set versus generalization. In networks with atypical computational properties, generalization (transfer to novel items) needed additional support from intervention items selected to highlight implicit regularities in the domain, regularities that typical networks could extract from normal experience. Atypical networks often best generalized through interpolation rather than extrapolation, since their properties could not support processing of items very different from those previously encountered.^6^

The idea of compensation arose in several contexts, and it is worth distinguishing the differences senses in which it was used. First, we saw one principled way to define compensation, by contrasting it with normalization (Yang & Thomas, 2015). In *normalization*, the aim of intervention is to provide the full range of abilities and knowledge that any typically developing system acquires through exposure to the normal training set. In this sense of *compensation*, the aim of the intervention is to optimize a subset of behaviors present in the original training set. Other models provided alternative senses of a ‘compensated’ system. These were forcing a system to find a partial solution to the cognitive domain through overtraining, but leaving residual deficits; and recruiting other mechanisms to deliver the same or similar behavior. These three senses would translate to three distinct approaches to intervening upon an atypical system: (a) selecting an intervention that targets a subset of the target cognitive domain; (b) providing greater practice to force greater accuracy from an atypical system, or simply leaving the system to improve through more experience; (c) employing explicit strategies to encourage the use of alternative mechanisms.

#### Modeling limitations

A key aspect of building models is simplification. We should be clear, then, the ways in which the computational work we have reviewed falls short with respect to the practice of interventions for developmental disorders.

On a broader scale, a focus on cognitive mechanism does not capture the complexity of the intervention situation, which can depend on dynamics of the interaction between the child and the speech and language therapist, and where intervention is sometimes a process of discovery of what works for individual children in the context of their family and school environment. To some extent, even fairly mechanism-focused interventions involve substantial behavioral and interactional interchange between the children and the therapist (and parent, if also coached), which may yield collateral benefits. Simulations do not address some of the complexities, such as distinguishing the effects of explicit instruction from implicit, the role of the expertise of the therapist, the effects of adaptive versus nonadaptive instruction, the distinction between 1-to-1 versus group instruction, the difference between therapist-delivered and parent-delivered interventions. Moreover, as Beauchaine et al. argue: “opponents of biological approaches to prevention and intervention also argue that by emphasizing genetic and neurobiological processes, we divert attention and resources away from important psychosocial causes of maladjustment, such as stress, parenting, and family interactions” (Beauchaine, Neuhaus, Brenner, & Gatzke-Kopp, 2008, p.748). Work in the implementation sciences has also pointed to wider limiting, enabling, and incentivizing factors for changing behavior beyond cognitive mechanisms, such as resources and policy (e.g., Michie et al., 2011).

On a narrower scale, our focus was on a limited set of computational architectures: associative networks. It is possible that other architectures used in cognitive models, such as self-organizing maps or attractor networks, might provide different plasticity conditions or effects of intervention on generalization. These remain to be explored. The observation that interventions for different language skills required different levels of intensity, duration, and interleaving (Lindsay et al., 2010) is consistent with the view that different types of mechanism are in play. Speculatively, it may be that intensity is more important than duration to change sensory representations (self-organizing systems); that repeated short bursts over an extended time are necessary to alter access to representations (associative systems); and that an extended duration of practice is necessary to extract regularities in complex sensorimotor sequences (recurrent networks). In addition to different architectures, it is necessary to consider control systems, mechanisms of executive function and reward-based learning, to address the origin and malleability of deficits in behavioral regulation, such as the restricted repertoire of interests in autism, or attentional deficits in Fragile X syndrome, or impulsivity in ADHD. Lastly, the model framework captures development in terms of a plastic mechanism exposed to a structured learning environment. However, this does not readily lend itself to considering the possibility that the disorder may change the structure of the learning environment via indirect pathways. For example, poor reading levels may reduce the child’s motivation to spend time reading, or parents may respond differently to children with learning disabilities than they would typically developing children.

As with Plaut’s (1996) influential connectionist model examining relearning following acquired damage, we took a simplifying step of first adopting a single mechanism perspective. However, behavior is generated by the interaction of multiple mechanisms. A multiple-mechanism framework is necessary to consider, variously, interventions to encourage alternative strategies, the use of executive function skills to compensate for weaknesses in domain-specific systems (Johnson, 2012), and interventions that might address deficits in functional connectivity between mechanisms (e.g., as sometimes proposed as a key deficit in autism; see Thomas et al., 2016, for discussion). The Best et al. (2015) model holds some promise in this regard, because it captures separate behaviors stemming from the operation of components (nonword repetition, semantic categorization) and from the interaction between components (naming, comprehension), where each behavior exhibits its own developmental trajectory. Within such a multiple-mechanism framework, it is apparent that a single mechanism can nevertheless serve as a limiting factor on performance, even if it is not the sole generator of behavior.

#### Integrating models with data from cognitive neuroscience

Neuroanatomically constrained models of the reading system and the semantic system have indicated how paying attention to neuroscience data can progress computational modeling and provide a paradigm for the modeling of intact and impaired cognitive abilities (e.g., Chen et al., 2017; Lambon Ralph, Jefferies, Patterson, & Rogers, 2017; Ueno et al., 2011). This work brought together models of normal processing of tasks such as word and object naming, detailed behavioral profiles from a large cohort of patients, and facts about the nature of the underlying impairment that could be related to properties the computational models, which together could explain a wide range of facts about deficit patterns, bases of recovery of function, and responsiveness to intervention. Each component—modeling, behavioral evidence, brain evidence—helped to bootstrap the other. The models suggested new ways of looking at brain and behavior, but the brain evidence also constrained how the impairments were simulated, yielding new testable predictions. These models incorporate multiple components and pathways, and simulate several target behaviors (e.g., for the reading model, repetition, comprehension, and naming). They have been applied to the simulation of acquired deficits, such as aphasias, semantic dementia, and visual agnosia, by removing connections from certain regions of the model, while retraining the model after damage has then allowed investigation of plasticity related recovery. Models of developmental deficits and interventions are less well progressed, but ideally would develop in the same direction (Woollams, 2013). What cognitive neuroscience data could be used to constrain such computational models?

There is a fast-growing literature identifying differences in brain structure and function in children with behaviorally defined developmental disorders. These include differences in global brain structure (e.g., reduced global gray matter in ADHD, Batty et al., 2010; increased brain size in autism, Waldie & Saunders, 2014); differences in local brain structure (e.g., thinner cortex in the pars opercularis in ADHD, a region involved in inhibitory control, Batty et al., 2010; smaller amygdala in children with Oppositional Defiant Disorder [ODD] and Conduct Disorder [CD], a region involved in emotion processing, Noordermeer, Luman, & Oosterlaan, 2016); and structural connectivity (e.g., abnormal anatomy of fronto-striatal white matter tracts in autism; Langen et al., 2012). Research using functional MRI indicates that in disorders, activation can be either reduced or increased in relevant areas, or increased in other areas. For example, in developmental dyslexia, within the normal reading network, the left temporo-parietal region and ventral occipito-temporal region are often underactivated, while the left inferior frontal gyrus is sometimes overactivated as a result of compensatory articulatory effort, whereas some studies also report increased activation outside the reading network in the right hemisphere (Barquero, Davis, & Cutting, 2014). Functional connectivity is sensitive both to individual differences (e.g., in working memory, in the link between the fronto-parietal network and visual areas; Barnes, Woolrich, Baker, Colclough, & Astle, 2016) and to disorders (e.g., abnormal resting state cortical connectivity between frontal and posterior regions in autism; Waldie & Saunders, 2014).

There are two kinds of challenge in adapting these new neuroanatomically constrained architectures to developmental disorders. The first challenge is to identify the relevant computational deficit to apply to one or more regions of the architecture, in this case prior to development rather than in a trained model for acquired deficits. For example, Seidenberg (2017) identified several candidate neural deficits that might be associated with developmental dyslexia, broadly falling under the view that signal propagation between and within regions is noisier (Hancock et al., 2017). These include greater variability in neural responses to stimuli, consequent reduced functional connectivity between regions, and slower learning from experiences. Implicated in noisier signaling are potential disruptions to myelination, changes to neural dynamics (hyperexcitability), and anomalies in neural migration. This is quite a wide set of computational anomalies, which in implemented models could have diverse effects on development and diverse responses to intervention.^7^

The second challenge is to determine how to intervene on larger, interactive architectures. As we have seen, in architectures with multiple mechanisms and pathways, there is the scope for alternate routes to compensate for anomalies in a given component. This indeed is what occurs during relearning after focal removal of connections to capture rehabilitation (Ueno et al., 2011). However, in a developmental deficit, the system is presumed to be plastic throughout, and the question arises as to why such compensation would not have taken place already. What intervention procedure could trigger reorganization in a way that natural experience could not? Perhaps it is as simple as giving extra practice on behaviors most closely linked to those brain regions showing reduced activation, such as phonological awareness training for temporal regions processing phonology in the case of dyslexia. Once more, preceding results caution us that even in this simple case, there may be timing effects, such that unless a narrow locus of developmental deficit is remediated early, the rest of the system may not be able to adjust without additional intervention. And of course, in larger architectures, deficits need not be focal, they could be widespread, or have spread across development from an initially more restricted locus.

Cognitive neuroscience can also provide data on response to intervention. In many cases, behavioral intervention leads to increased activation in previously underactivated regions and changes in functional connectivity that bring individuals closer to the patterns observed in typically developing controls, so-called normalization (e.g., in dyslexia: Ylinen & Kujala, 2015; in autism: Calderoni et al., 2016; Waldie & Saunders, 2014). However, sometimes individuals respond to intervention with decreased activation or compensatory recruitment of different regions, and regions that respond to intervention are often not localized but widespread across the brain. It is an area of active research to uncover whether such neural markers can predict how individual children respond to intervention (Barquero et al., 2014). In one study of reading deficits, Simos et al. (2007) found that children who responded to intervention exhibited normalization while nonresponders exhibited compensation.

Overall, research from neurodevelopment exhibits similar themes to the computational modeling work described here—contrasting normalization with compensation, identifying individual differences in response to intervention, distinguishing resolving from persisting delays, interpreting the implications of good compensatory outcomes. However, the neuroscience literature is also very mixed—in part because of heterogeneity in methods, in part because of heterogeneity in participants. For example, a difference in one direction between disorder and control group in one study may be contrasted by a difference in the opposite direction in another (e.g., in the size of the amygdala in ODD and CD, Noordermeer et al., 2016, for review; in the activation of inferior frontal gyrus in dyslexia, Barquero et al., 2014, for review). Patterns of brain responses to intervention can be complex. The logic of linking activation or structure to behavior is not always clear: to remediate a behavioral deficit, is more activation or less activation better? Is thinner cortex or thicker cortex better? Is more connectivity or less connectivity better? Karmiloff-Smith (2010) argued that for brain imaging to advance our understanding of development, it has to focus on mechanisms of change, rather than static snapshots of structural or functional properties. The computational models we have considered are orders of magnitude simpler than real neural systems. Yet they generate a vocabulary to consider how mechanisms of change may cause atypical development and constrain response to intervention. As we saw with attempts to link Thomas and Knowland’s (2014) notions of capacity and plasticity to brain properties, the continuing challenge is to drive closer links between cognitive models and brain systems.

### Conclusion: The Importance of Narrowing the gap

Advances in mechanistic, computational models of developmental disorders (and more widely, individual variability) set the foundation for an investigation of intervention. Implementation can provide a driver for advances in theory, although questions remain about whether the simplification necessary for modeling omits key dimensions of the intervention situation, notably its usual basis in social interaction. It is important to narrow the gap between theories of deficit and theories of intervention, to place intervention on an evidence-driven, mechanistic basis. Practice-based approaches naturally emphasize behavioral consequences of intervention and are less focused on understanding mechanisms: for these approaches, what is important is what works behaviorally and what can enable success. This emphasis on proximate goal is one of the reasons for the gap. However, understanding the active agent underpinning a successful intervention is key to understanding what will work in which contexts for what disorders, as well as the flexibility of the application of a given technique (Law et al., 2008). As Nathan and Wagner Alibali (2010) argue, to narrow the gap, we need a combination of scaling-up from the elemental, mechanistic models of cognitive science and scaling-down from the complexity of real-life intervention situations. That in turn requires clinicians to be interested in mechanism, despite it being an understandably lower priority than behavioral outcomes for the children they treat.

## Figures and Tables

**Table 1 tbl1:** Key Concepts

Cause of disorder	Intervention outcomes	Interventions in developmental models	Types of simulated interventions
Monogenic (single cause)	Does the intervention generalize beyond the treated items to other items or skills?	Does intervention improve performance on the training set?	Behavioral (add new items to/change frequency distribution of training set)
Polygenic (multiple causes)	Is there maintenance of gains after the intervention ceases?	Does intervention improve performance on the generalization set (novel items)	Computational (alter the computational properties of the learning mechanisms)
			Compensatory (encourage alternate mechanisms/pathways to acquire target behaviors)

**Table 2 tbl2:** Use of Internal Computational Parameters to Predict Developmental Outcomes (Persisting Delay, Resolving Delay) in a Polygenic Model of Language Delay

Computational parameter	Processing role	Effect size of PD versus RD comparison
Number of internal units	Capacity	.031**
Pruning threshold	Capacity/Regressive events	.021*
Learning algorithm	Capacity/Plasticity	.104**
Lexical-semantic learning rate	Plasticity	.024**
Unit discriminability	Plasticity/Signal	.025**
Processing noise	Signal	.026**
*Note*. PD = persisting delay; RD = resolving delay. Scores show η_p_^2^ effect sizes from ANOVA comparing PD and RD groups (see Thomas & Knowland, 2014, Table 2, for parallel analyses using logistic regression methods).		
* Effect reliable at *p* < .05. ** Effect reliable at *p* < .01.		

**Table 3 tbl3:** A Simulated Intervention That Produced Different Effects on Population Mean Performance and Standard Deviations, Depending on Timing and Target Behavior

Measure	Early intervention (epoch 50) mean population accuracy and variation								
Epoch	25	50	55	60	75	100	250	1000	
Epoch postintervention			+5	+10	+25	+50	+150	+950	
Regular verbs									
Untreated									
*Mean*	.47	.60	.61	.62	.65	.67	.73	.75	
*SD*	.29	.27	.27	.27	.26	.26	.25	.23	
Treated									
*Mean*			.67	.73	.81	.86	.94	.97	
*SD*			.22	.19	.14	.11	.07	.05	
Irregular verbs									
Untreated									
*Mean*	.07	.15	.17	.19	.23	.27	.41	.49	
*SD*	.07	.13	.14	.15	.17	.19	.23	.26	
Treated									
*Mean*			.13	.16	.24	.36	.64	.80	
*SD*			.13	.15	.17	.20	.23	.22	
Late intervention (250 epochs) mean population accuracy and variation									
Irregular verbs									
Epoch	250	255	260	275	300	350	500	750	1000
Postintervention		+5	+10	+25	+50	+100	+250	+500	+750
Untreated									
*Mean*	.41	.41	.41	.42	.43	.44	.46	.48	.49
*SD*	.23	.23	.23	.24	.24	.24	.25	.26	.26
Treated early									
*Mean*		.64	.64	.66	.67	.70	.75	.79	.80
*SD*		.23	.23	.23	.23	.23	.23	.22	.22
Treated late									
*Mean*		.34	.34	.37	.41	.46	.55	.60	.63
*SD*		.24	.25	.26	.27	.28	.29	.30	.31
*Note*. A population of 1,000 networks learning English past tense experienced an intervention either early (after 50 epochs) or late (250 epochs) in development. During intervention, differences in the richness of the environment between individuals were removed and all networks given the most enriched training set. Early intervention improved the population mean for regular verbs and reduced variation attributable to ceiling effects. Early intervention improved population mean for irregular verbs but did not alter variation—gaps between individuals did not narrow. Late intervention improved population mean for irregular verbs (though less so than early intervention) but increased population variation—gaps between individuals widened after intervention.									

**Table 4 tbl4:** Standardized Beta Values for Linear Regressions Predicting Individual Differences in Treatment Effect Sizes Following Two Different Types of Intervention, Normalization and Compensation, in Simulated Networks With a Connectivity Over-Pruning Disorder (Davis, 2017)

	Intervention type			
	Normalization		Compensation	
Parameter	Training set performance	Generalization performance	Training set performance	Generalization performance
Number of hidden units	−.016	.012	.011	.023
Sigmoid temperature	−.040	−.001	**−.098**	**−.127**
Processing noise	.028	.032	.007	−.012
Learning rate	**−.065**	**−.086**	−.053	−.016
Momentum	−.014	−.011	−.013	−.011
Initial weight variance	−.015	−.002	−.031	−.023
Architecture	**−.110**	**−.101**	**−.112**	**−.092**
Learning algorithm	−.006	−.059	−.011	.010
Response threshold	−.055	−.063	.000	.036
Pruning onset	.022	−.007	.057	.045
Pruning rate	−.006	.006	−.062	**−.075**
Pruning threshold^a^	.014	**.082**	.039	−.047
Weight decay rate	.021	.007	.036	.025
Sparseness of connectivity	.027	**.065**	.049	.052
Richness of environment	−.030	−.036	−.028	−.028
*Note*. *N* = 790 networks (only those from the population showing a behaviorally assessed performance deficit). Separate regressions were carried out for performance on the training set and generalization set. The shaded area shows parameters related to the pathological process, elevated values of the pruning threshold, permitting larger connections to be removed following the onset of pruning. Bold shows significant at *p* < .05.				
^a^ This parameter was set to atypical values to produce the developmental disorder.				

## Footnotes

1 The choice of parameters to vary was based on previous connectionist models that had used individual parameter variations to explain individual differences or disorders. These models were pursuing hypotheses that, for instance, differences in cognition may arise from neural plasticity or from the actions of certain neurotransmitters. Variations in *architecture* have been used to explain dyslexia: Zorzi, Houghton, and Butterworth (1998); in *hidden units* to explain intelligence: Richardson, Baughman, Forrester, and Thomas (2006), Richardson, Forrester, Baughman, and Thomas (2006) and autism: Cohen (1998); in *sparseness of connectivity* to explain autism: McClelland (2000); in *processing noise* to explain DLD: Joanisse and Seidenberg (2003); in *unit threshold function* to explain schizophrenia: Cohen and Servan-Schreiber (1992) and aging: Li and Lindenberger (1999); in *connection pruning* to explain autism: Thomas et al. (2011); and in *learning rate* to explain general intelligence: Garlick (2002). Here, individual differences were produced by simultaneous small variations in all parameters.

2 Predictive power is lost because of the interactions between the computational parameters in such mechanisms, where many of the effects are nonlinear (Thomas et al., 2016).

3 In an equivalent population of 1000 networks without atypical pruning, the number of connections in a network correlated .134 with behavior at epoch 250 and .163 with behavior at epoch 1000 (both *p* < .001).

4 For example: https://www.nih.gov/news-events/news-releases/brain-activity-pattern-signals-ability-compensate-dyslexia, retrieved 17 August 2016: “Understanding the brain activity associated with compensation may lead to ways to help individuals with this capacity draw upon their strengths. Similarly, learning why other individuals have difficulty compensating may lead to new treatments to help them overcome reading disability” (Alan E. Guttmacher, M.D., director of the NIH’s Eunice Kennedy Shriver National Institute of Child Health and Human Development, commenting on Hoeft et al., 2011).

5 The enrichment intervention, described in Figure 3, was applied to the Thomas and Knowland (2014) model, and trajectories of response to intervention were traced separately for those whose early identified delay (if untreated) would resolve versus those where it would persist. The maximally enriched training set was applied to all networks at epoch 50. For the following 30 epochs, resolvers and persisters improved by the same amount. Thereafter, resolvers (*N* = 165) showed a faster rate of improvement than persisters (*N* = 64; epoch × group interaction, *F*[1,227] = 5.06, *p* = .025, η_p_^2^ = .022), so that there was a reliable difference in treatment effect between the groups by 80 epochs post-onset of intervention (5.6% improvement in accuracy for resolvers, 3.4% improvement for persisters, *t*[227] = 2.56, *p* = .011).

6 Plaut (1996) found that simulated recovery of reading following acquired damage was better supported by retraining on atypical semantic category members than prototypical category members. This can be seen as an example of encouraging training transfer by interpolation. In Plaut’s implementation, atypical category members surrounded prototypical category members in semantic space. Training on the surrounding members transferred to those lying in between.

7 Currently, no straightforward behavioral intervention follows from the neural noise hypothesis of developmental dyslexia. Hancock et al. (2017) argue that the hypothesis points to interventions via brain-stimulation techniques, such as transcranial direct current stimulation and transcranial magnetic stimulation, or pharmacological agents, to address the hypothesized hyperexcitability of neurons.
